# Evaluation of the Induction of Immune Memory following Infant Immunisation with Serogroup C *Neisseria meningitidis* Conjugate Vaccines – Exploratory Analyses within a Randomised Controlled Trial

**DOI:** 10.1371/journal.pone.0101672

**Published:** 2014-07-14

**Authors:** Ameneh Khatami, Elizabeth A. Clutterbuck, Amber J. Thompson, Jennifer A. McKenna, David Pace, Jacqueline Birks, Matthew D. Snape, Andrew J. Pollard

**Affiliations:** 1 Oxford Vaccine Group, Department of Paediatrics, University of Oxford and the NIHR Oxford Biomedical Research Centre, Oxford, United Kingdom; 2 Malta Children's Vaccine Group, Mater Dei Hospital, Tal-Qroqq, Msida, Malta; 3 Centre for Statistics in Medicine, University of Oxford, Oxford, United Kingdom; Public Health England, United Kingdom

## Abstract

**Aim:**

We measured meningococcal serogroup C (MenC)-specific memory B-cell responses in infants by Enzyme-Linked Immunospot (ELISpot) following different MenC conjugate vaccine schedules to investigate the impact of priming on immune memory.

**Methods:**

Infants aged 2 months were randomised to receive 1 or 2 doses of MenC-CRM_197_ at 3 or 3 and 4 months, 1 dose of MenC-TT at 3 months, or no primary MenC doses. All children received a *Haemophilus influenzae* type b (Hib)-MenC booster at 12 months. Blood was drawn at 5, 12, 12 months +6 days and 13 months of age.

**Results:**

Results were available for 110, 103, 76 and 44 children from each group respectively. Following primary immunisations, and prior to the 12-month booster, there were no significant differences between 1- or 2-dose primed children in the number of MenC memory B-cells detected. One month following the booster, children primed with 1 dose MenC-TT had more memory B-cells than children primed with either 1-dose (p = 0.001) or 2-dose (p<0.0001) MenC-CRM_197_. There were no differences in MenC memory B-cells detected in children who received 1 or 2 doses of MenC-CRM_197_ in infancy and un-primed children.

**Conclusions:**

MenC-specific memory B-cell production may be more dependent on the type of primary vaccine used than the number of doses administered. Although the mechanistic differences between MenC-CRM_197_ and MenC-TT priming are unclear, it is possible that structural differences, including the carrier proteins, may underlie differential interactions with B- and T-cell populations, and thus different effects on various memory B-cell subsets. A MenC-TT/Hib-MenC-TT combination for priming/boosting may offer an advantage in inducing more persistent antibody.

**Trial Registration:**

EU Clinical Trials Register 2009-016579-31

ClinicalTrials.gov NCT01129518

## Introduction

As a result of the sustained increase in serogroup C meningococcal (MenC) disease in the United Kingdom (UK) in the 1990's, three MenC conjugate vaccines were licensed and introduced into the routine infant immunisation schedule. These included two different vaccines conjugated to a mutant diphtheria toxoid (CRM_197_), and one conjugated to tetanus toxoid (TT). MenC conjugate vaccines induce bactericidal polysaccharide-specific antibodies, which have been shown to correlate with protection against invasive disease [1], [2], [3]. In addition to inducing immunological memory, as defined by an anamnestic antibody response to subsequent challenge, several different factors are thought to contribute to long-term protection after immunisation with conjugate vaccines including reduced carriage, herd immunity and persistence of bactericidal antibody in the serum [4], [5].

In the UK, the currently available MenC-CRM_197_ and MenC-TT conjugate vaccines are used interchangeably in the immunisation schedule; however there is evidence that the TT-conjugated vaccine is more immunogenic and in particular is a better “priming” vaccine irrespective of the type of booster vaccine that is subsequently administered [6], [7]. Furthermore, higher serum bactericidal assay (SBA) titres were observed following *Haemophilus influenzae* type b (Hib) and MenC conjugate (Hib-MenC-TT) booster in children primed with Hib-MenC-TT than children primed with monovalent MenC-CRM_197_ in the first year of life, even though post-primary immunisation SBA titres were lower in the former group [8], [9]. These findings may relate to differences in the ability of these vaccines to generate memory B-cells following primary immunisations. It has been shown that antibody levels following a MenC-TT booster at 12 months of age are higher in children who received 1 dose of the same vaccine at 4 months of age, compared to children who received 2 doses at 2 and 4 months [10], and that infants primed with 1 dose of MenC-TT mounted a greater antibody response to a polysaccharide challenge at 12 months of age, compared with those primed with either 2 or 3 doses of MenC-TT in infancy [11], suggesting that the number of doses of primary vaccines may also be important in the generation of memory B-cells.

Frequency of antigen-specific memory B-cells in peripheral blood can be quantified by Enzyme-Linked Immunospot (ELISpot) that detects immunoglobulin (Ig) G antibody secreting cells (ASCs). As part of a larger study, we investigated MenC-specific memory B-cells following different MenC conjugate vaccine schedules in infancy to determine the effect of the number of priming doses and type of vaccine on the number and kinetics of memory B-cells generated.

## Materials and Methods

The protocol for this trial and supporting CONSORT checklist are available as supporting information; see Checklist S1 and Protocol S1.

### Participants and Vaccines

Ethical approval for the study was granted by the Oxfordshire Research Ethics Committee (study number: OXREC 10/H0604/7). Participants included in the data reported here were enrolled in a large open-label randomised controlled trial conducted in four centres in the UK and one centre in Malta. Children recruited at a research site with access to the necessary laboratory facilities, who were able to provide sufficient volumes of blood (>4 mL), and where samples were able to be processed within 6 hours were included in the analysis reported here. Previously un-immunised, healthy term infants aged 6–12 weeks, without evidence of pre-existing medical conditions were enrolled by study doctors when written informed consent was obtained from parents or guardian. Participants were randomised to one of four study groups by sequentially opening sealed envelopes prepared by an independent statistician. The ratio of children randomised to each group was 10∶10∶4∶7 respectively for 1-dose MenC-CRM group: 2-dose MenC-CRM group: 1-dose MenC-TT group: control group. Randomisation was generated in fixed blocks of 30 using Stata version 10 (StatCorp LP, Texas, USA). Participants in the 1-dose MenC-CRM group received MenC-CRM_197_ (*Menjugate Kit*, Novartis Vaccines and Diagnostics, Basel, Switzerland) at 3 months of age; the 2-dose MenC-CRM group received MenC-CRM_197_ at 3 and 4 months of age; the 1-dose MenC-TT group received MenC-TT (*Neisvac-C*, Baxter Healthcare, Deerfield, Illinois, USA) at 3 months; the control group did not receive any primary MenC vaccine doses. All children received a Hib-MenC-TT (*Menitorix*, GlaxoSmithKline Biologicals, Rixensart, Belgium) booster at 12 months of age.

Concomitant vaccines administered according to the routine UK infant immunisation schedule were: combination diphtheria, tetanus, acellular pertussis, inactivated poliovirus and Hib vaccine (*Pediacel*, Sanofi Pasteur MSD, Lyon, France) at 2, 3 and 4 months; 13 valent pneumococcal conjugate vaccine (*Prevenar-13*, Wyeth Vaccines, Pearl River, New York, USA) at 2, 4 and 12 months of age; and a combined measles, mumps and rubella vaccine at 13 months of age.

### Blood sampling

Blood was drawn at 5 months (1 or 2 months after primary immunisations), 12 months (before Hib-MenC-TT booster) and 13 months of age (1 month post-booster). All children in the control group, as well as an equal number from each of the other study groups also had a blood sample drawn 6 days after the 12 month Hib-MenC-TT booster. Study staff performing blood draws were not blinded to group assignment as they needed to know which vaccines to administer to each participant as part of the larger clinical trial.

### Separation of PBMCs by density gradient centrifugation

Heparinised whole blood samples (2–4.5 ml) were processed within 6 hours of collection. PBMCs were isolated by diluting blood in R0 “complete” medium and layering over a density gradient medium (Lymphoprep; Alere, UK) as previously described by Kelly *et al*, and Blanchard *et al* [12], [13].

### *In vitro* stimulation of PBMCs for differentiation of memory B-cells into ASCs

2×10^5^ cells/well of isolated PBMCs were seeded into 96-well, cell culture treated plates (Greiner-One Bio, UK) and stimulated with 100 µl/well of a mixture of 1∶5000 *Staphylococcus aureus* Cowan 1 strain (SAC Pansorbin cells, Merck-Millipore, UK), 1.7 µg/ml CpG (BioScience Ltd, UK) and 83.33 ng/ml pokeweed mitogen (Sigma-Aldrich, UK). This combination induces polyclonal stimulation of B-cells and maximal proliferation of memory B-cells allowing differentiation of small antigen-specific populations into IgG-ASCs that may be detected by ELISpot [14], [15]. Plates were incubated at 37°C, 5% CO_2_ and 95% humidity for 5–6 days. Harvested cells were washed in phosphate buffered saline with ethylenediaminetetraacetic acid (EDTA) di-sodium and 0.5% newborn bovine serum (Sigma-Aldrich, UK) and re-suspended in R10 medium to a concentration of 2×10^6^ cells/ml.

### Preparation of antigen coated ELISpot plates

96-well multiscreen-IP filter plates with polyvinylidene membranes (Merck-Millipore, UK) were coated with 100 µL the following antigens: 10 µg/ml goat anti-human Ig (Caltag Medsystems, UK); 10 µg/ml diphtheria toxoid (Staten Seruminstitut, Denmark); 5 µg/ml tetanus toxoid (Statens Seruminstitut, Denmark); or 5 µg/ml MenC polysaccharide mixed with 5 µg/ml methylated human serum albumin (National Institute for Biological Standards and Control [NIBSC], UK). Anti-human Ig, tetanus and diphtheria toxoids were included as positive controls and 100 µL phosphate buffered saline (PBS) (pH 7.2–7.4) as background control.

### ELISpot assay for detection of IgG-ASCs

As previously described [12], [13], 2×10^5^ cells/well cultured PBMCs and 1∶100 and 1∶1000 dilutions of Ig controls were added to the antigen-specific wells and Ig-coated wells respectively. Plates were incubated at 37°C, 5% CO_2_ and 95% humidity overnight. The supernatants were then discarded and the plates were repeatedly washed with PBS and detergent (0.25% Tween). Plates were then incubated for 4 hours at room temperature with 50 µL/well (1∶5000) of goat anti-human γ-chain-specific alkaline phosphatase conjugate (Merck Chemicals Ltd, UK) and then washed repeatedly in PBS-Tween0.25% and sterile H_2_O. Spots were developed using 5-bromo-4-chloro-3-indolyl phosphate in nitroblue tetrazolium dissolved in aqueous dimethylformamide prepared from a kit (Bio-Rad Laboratories, UK).

### Automated enumeration of IgG-ASC spots

ELISpot plates were scanned and counted using AID ELISpot reader version 5.0 and verified by visual inspection. Identical settings were used for all plates and antigens by a blinded operator. If <3 well replicates were available and variation was more than 15%, the sample was excluded from analysis for that antigen. For samples taken from children aged 12 months or older, if the total IgG spots/million PBMCs was <1000, the sample was excluded from analysis. Due to the overall poor immune responses expected in infants, no exclusion criteria was used on the basis of a positive control (anti-IgG) response for blood samples drawn from children at 5 months of age. Other authors have used similar exclusion criteria when using the ELISpot assay to detect memory B-cells in blood from children >12 months of age [12], [16], but not used such an exclusion criteria for blood samples drawn from infants <12 months of age [13].

### Statistics

Sample size calculation for the full study was based on the primary objective to determine non-inferiority of antibody response following booster vaccination in the 1-dose MenC-CRM group compared to the 2-dose MenC-CRM group (to be reported elsewhere). The subset of participants on whom memory B-cells were measured were selected pragmatically from those recruited at the Oxford research site, from whom a sufficient blood volume could be drawn and where the sample could be processed in time. All comparisons of B-cell responses between study groups were exploratory. Primary analyses were based on all available blood samples (intention-to-treat analysis) as pre-specified in the statistical analysis plan for the randomised controlled trial within which this study was embedded. Calculations were carried out using Stata version 12 (StatCorp LP, Texas, USA) and GraphPad PRISM version 4.00 for Windows (GraphPad Software, San Diego California, USA, www.graphpad.com).

When no cells were detected in the assay for an antigen, results were assigned a value of half the lowest level of detection (0.31 cells/million PBMCs) to enable log_10_ transformation. A one-way analysis of variance (ANOVA) was conducted of the log_10_ transformed data to test for differences between groups in the number of memory B-cells detected at each visit. If the F-test for groups from the ANOVA was significant, pairs of groups were compared. Residual variance was checked for normality, and within-groups variance was tested for heterogeneity (Bartlett's test). If the data appeared to be non-normal, non-parametric Kruskal Wallis analysis was carried out. Comparisons were reported with 95% confidence intervals (CIs) and Bonferroni adjusted p-values for multiple comparisons.

Linear mixed models were used to estimate mean differences between two time-points for each group using log_10_ transformed data. The mixed model takes account of missing data and the correlation between measurements on the same child at two time-points. The fixed effects were: group, time-point and the interaction between group and time-point. The covariance matrix was unstructured. The mean difference between two time-points for each group was estimated using log_10_ transformed data.

## Results

**Figure 1:**
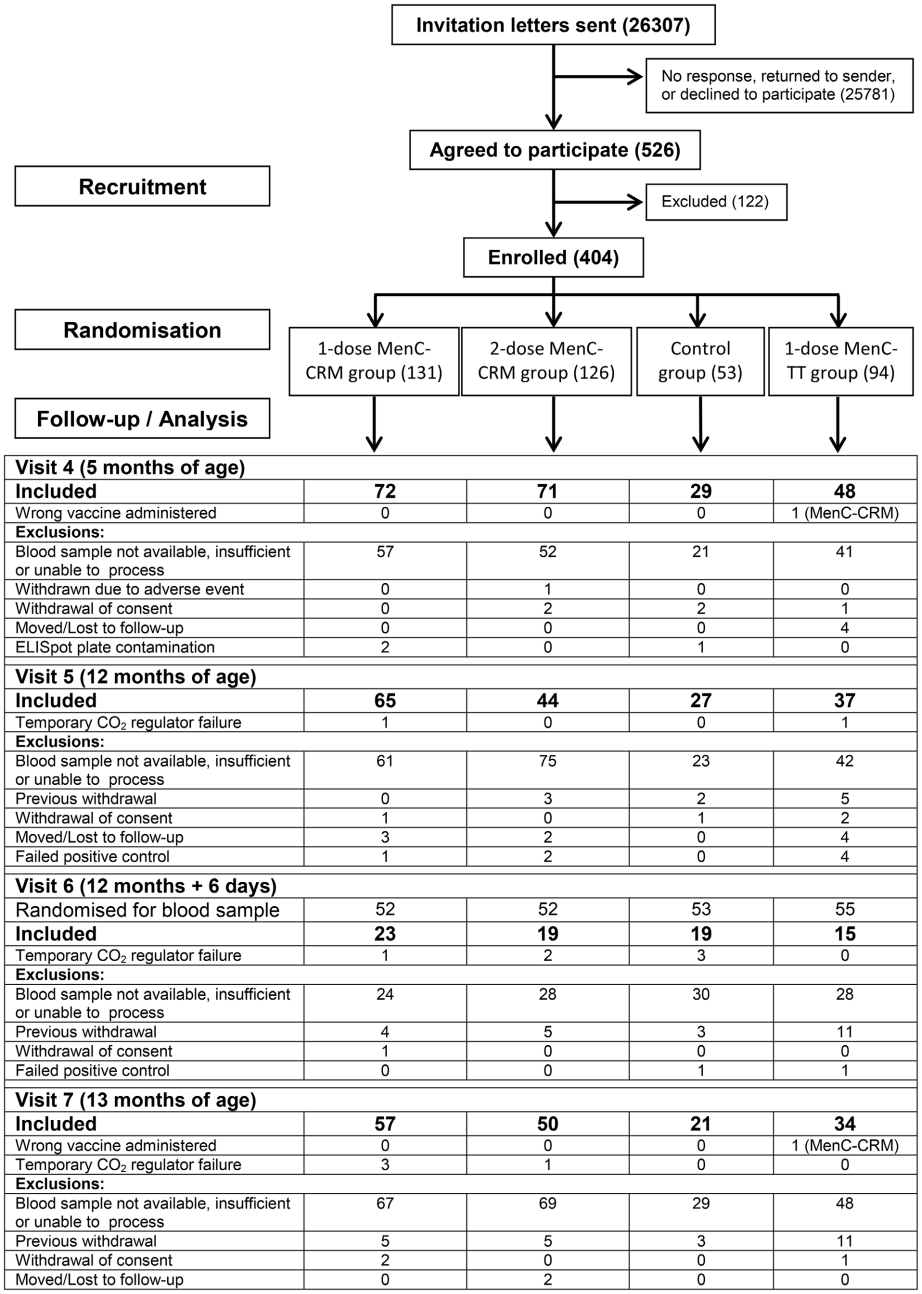
Number of children enrolled and included in the final analysis for MenC-specific memory B-cells.

**Figure 2:**
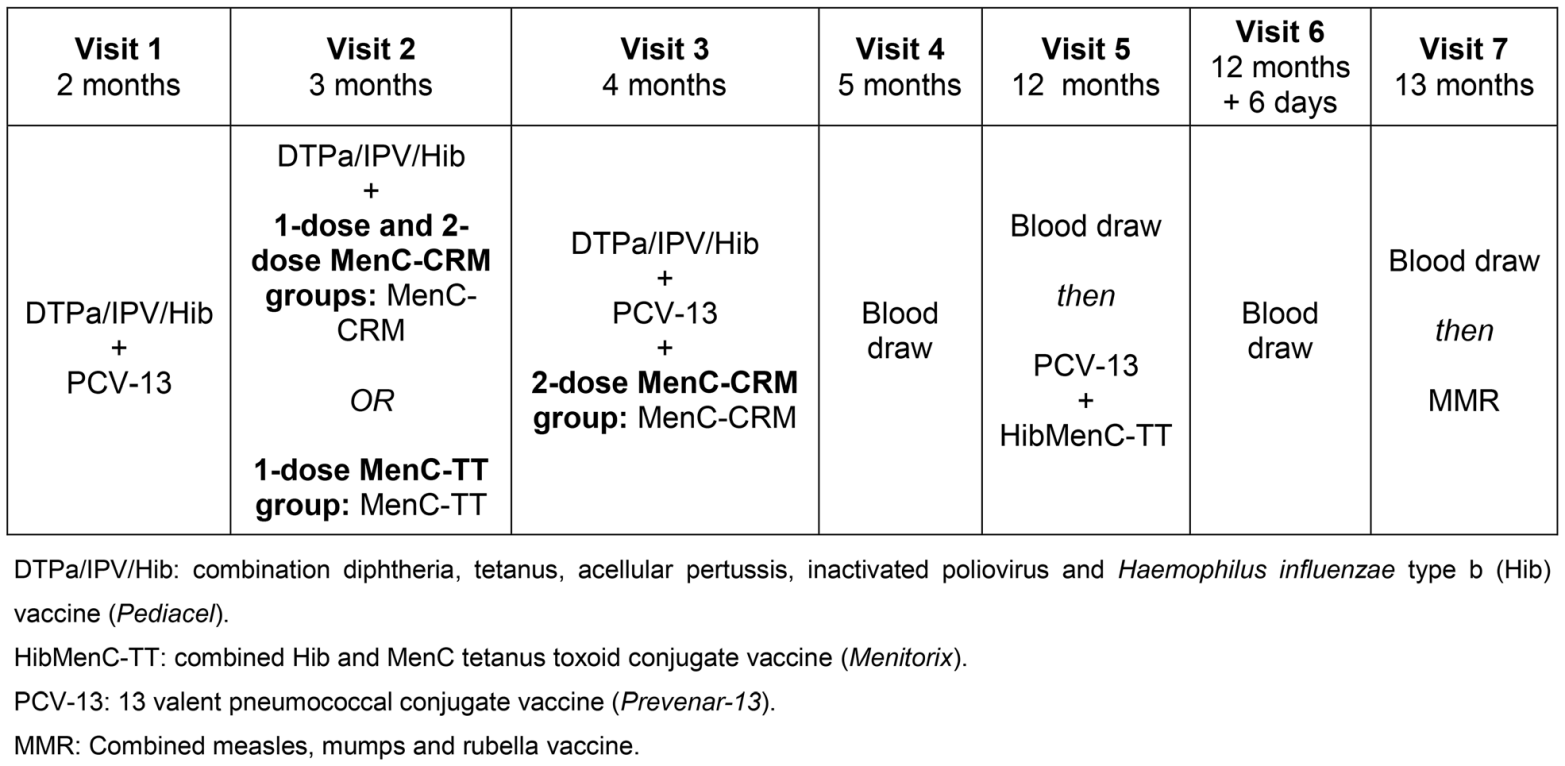
Schedule of study visits and procedures, including vaccines administered and timing of blood draws used to measure MenC-specific memory B-cells.

509 children were recruited across all research sites between July 2010 and July 2011, 404 of whom were recruited from a single site (Oxford) and included in the analysis presented here. Memory B-cell results were available from at least one time-point for 332 children, of whom 149 were female. 200 children had blood samples available for analysis from at least two time-points. The median age at first vaccination was 59 days (range 51–102). There were no significant differences between study groups with respect to age, gender or ethnicity of participants. 540 blood samples were available for analysis of memory B-cell responses. Details of sample inclusions and exclusions are outlined in figure 1. Figure 2 describes the study timeline with respect to vaccines administered and blood sampling time-points.

Nine samples, (2 from visit 5, 3 from visit 6 and 4 from visit 7) were affected by a temporary incubator CO_2_ regulator failure. These plates were placed into sealable plastic boxes with CO_2_ sachets when the failure was discovered, and results included in the final analysis. The duration of effect on each sample was between 3 to 6 days. One participant randomised to the 1-dose MenC-TT group received vaccinations according to the 1-dose MenC-CRM group. Results for this participant were analysed in the 1-dose MenC-TT group (intention-to-treat analysis). Sample exclusions and protocol deviations did not disproportionately affect any of the study groups.

### MenC-specific memory B-cells

**Figure 3:**
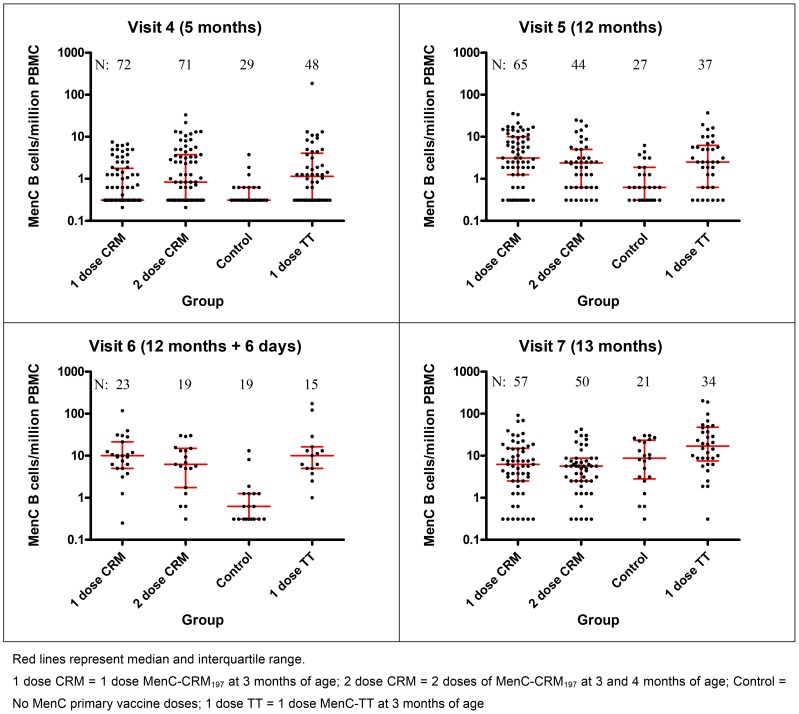
Number of MenC-specific memory B-cells (log_10_ scale) detected in the peripheral blood of individual participants after immunisation with different schedules of MenC conjugate vaccines, at each time-point following infant primary and booster vaccines.

Figure 3 illustrates all data points available for the number of MenC memory B-cells/million peripheral blood mononuclear cells (PBMCs) measured for each participant, according to study group, at each time-point.

At 5 months of age there were large numbers of samples in which no antigen-specific memory B-cells could be identified (46% of observations); the data were not normally distributed and were not analysed by parametric methods. No statistically significant difference was seen between children who received 1- or 2-dose MenC-CRM_197_ priming at any time-point in the number of MenC-specific memory B-cells generated (Table 1). Control children had fewer MenC memory B-cells than all other groups after the primary vaccines and until 1 month after the booster (table 1 and Table S1 in file S1). There were no statistically significant differences in the number of MenC memory B-cells generated one month after the Hib-MenC-TT booster by un-primed children (control group) and children who had received either 1 or 2 doses of MenC-CRM_197_ primary vaccines. By 13 months of age, children in the 1-dose TT group had generated more MenC-specific memory B-cells than children in either the 1-dose or 2-dose MenC-CRM groups (table 1).

**Table 1:** Comparison between groups of the log_10_ transformed number of MenC-specific memory B-cells detected in the peripheral blood at each visit.

		*Kruskal-Wallis statistic*	*p-value*
**5 months of age (1 or 2 months after primary vaccines)**			
Between groups 1-dose CRM, 2-dose CRM, and 1-dose TT		X^2^_2_ = 4.9	0.09
Groups 1-dose CRM, 2-dose CRM, 1-dose TT vs control*		X^2^_1_ = 10.2	**0.001**
	***F test and unadjusted p-value***	***Difference between groups with estimated 95% CI***	***Bonferroni adjusted p-value***
**12 months of age (pre-booster)**			
Between groups 1-dose CRM, 2-dose CRM, and 1-dose TT	F_2, 169_ = 1.4, p = 0.24		
Groups 1-dose CRM, 2-dose CRM, 1-dose TT vs control*	F_1, 169_ = 16.6, **p = 0.0001**		
**12 months +6 days (6 days after booster vaccination)**			
Between groups 1-dose CRM, 2-dose CRM, and 1-dose TT	F_2, 72_ = 1.2, p = 0.3		
Groups 1-dose CRM, 2-dose CRM, 1-dose TT vs control*	F_1, 72_ = 49.9, **p<0.0001**		
**13 months of age (1 month after booster vaccination)**			
1-dose CRM vs control	F_1, 158_ = 0.4, p = 0.51	−0.10 (−0.41 to 0.21)	1.0
2-dose CRM vs control	F_1, 158_ = 1.4, p = 0.25	−0.19 (−0.50 to 0.13)	1.0
1-dose TT vs control	F_1, 158_ = 6.0, p = 0.02	0.42 (0.08 to 0.765)	0.09
1-dose CRM vs 2-dose CRM	F_1, 158_ = 0.5, p = 0.5	0.08 (−0.15 to −0.32)	1.0
1-dose CRM vs 1-dose TT	F_1, 158_ = 15.2, p = 0.0001	−0.52 (−0.78 to −0.26)	**0.001**
2-dose CRM vs 1-dose TT	F_1, 158_ = 19.5, p<0.00001	−0.60 (−0.87 to −0.33)	**<0.0001**

1-dose CRM: 1 dose MenC-CRM_197_ at 3 months of age; 2-dose CRM: 2 doses of MenC-CRM_197_ at 3 and 4 months of age; Control: No MenC primary vaccine doses; 1-dose TT: 1 dose MenC-TT at 3 months of age.

*Results of the analysis of pairs of groups are provided in Table S1 in file S1.

Using linear mixed models to analyse the log_10_ transformed data, a statistically significant rise was seen in the number of MenC-specific memory B-cells between the pre- and post-booster blood samples for each study group with a greater rise seen in the control group and the 1-dose MenC-TT group compared to the 1- and 2-dose MenC-CRM groups (Table S2 in file S1). P-values are reported for the comparison of each group mean difference with 0.

### Frequencies of MenC-specific memory B-cells

The proportion of MenC-specific memory B-cells out of the total pool of IgG positive memory B-cells was calculated at each time-point for all the primed children (pooled samples from 1-dose MenC-CRM, 2-dose MenC-CRM and 1-dose MenC-TT groups) as well as for unprimed children in the control group (table 2). The median proportion of MenC-specific memory B-cells in the control group was 0% until 1 month after the Hib-MenC-TT booster. By 13 months of age 0.07% (median) of control children's IgG positive memory B-cells were MenC-specific, similar to the median percentage of MenC-specific memory B-cells in previously primed children (0.08%).

**Table 2:** Proportion of MenC-specific memory B-cells out of the total pool of IgG positive memory B-cells detected in the peripheral blood at each time-point.

		5 months of age	12 months of age	6 days after 12-month booster	13 months of age
**Primed children**	Median	0.02%	0.04%	0.12%	0.08%
	IQR	0%–0.097%	0.013%–0.094%	0.054%–0.22%	0.033%–0.23%
**Unprimed children (Control group)**	Median	0.0%	0.0%	0.0%	0.07%
	IQR	0%–0.016%	0%–0.022%	0%–0.019%	0.029%–0.20%

IQR: Interquartile range.

### Differentiating primed and un-primed children

**Figure 4:**
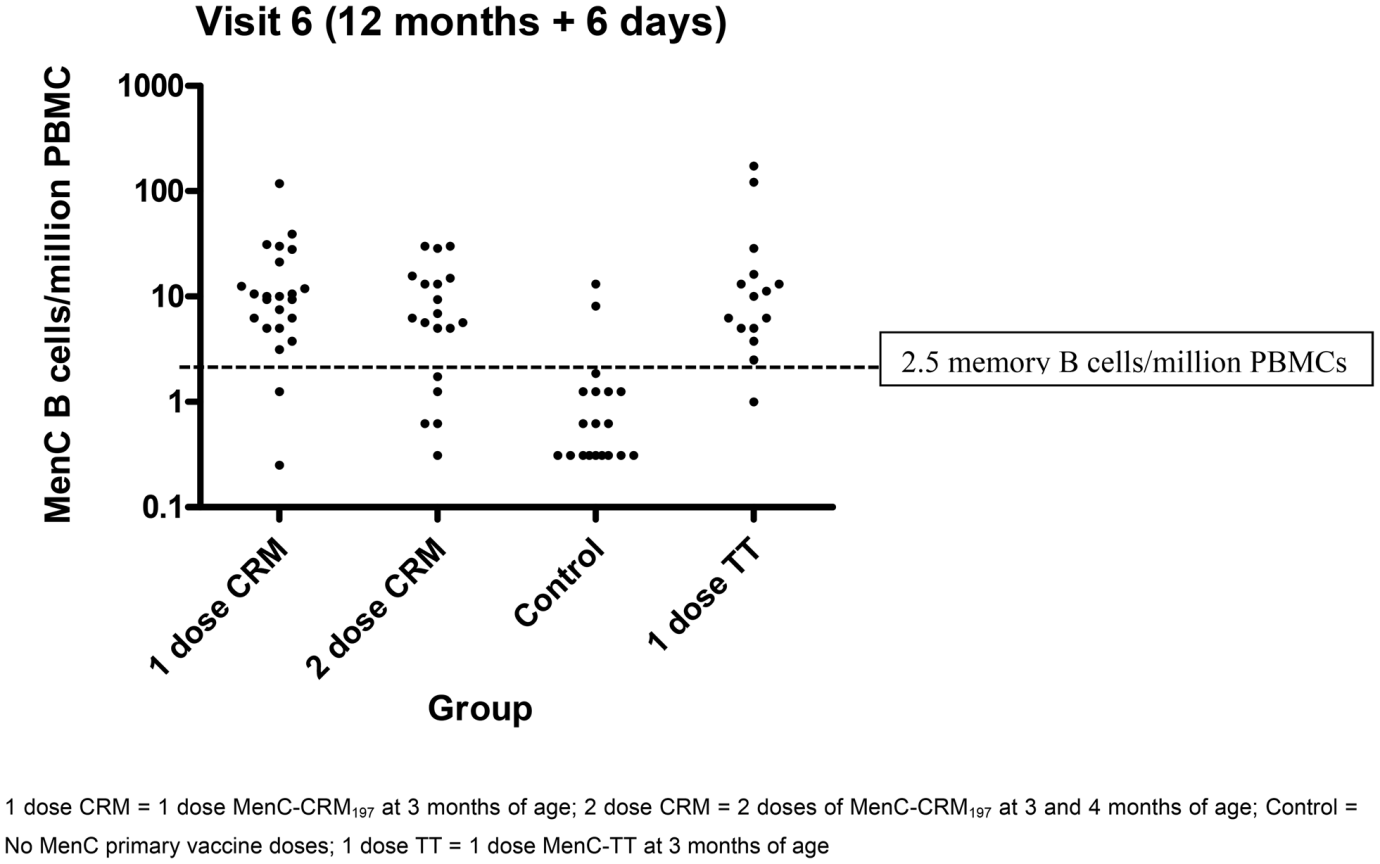
Number of MenC-specific memory B-cells detected in the peripheral blood of infants 6 days after a Hib-MenC-TT booster at 12 months of age, according to different primary immunisation schedules (magnified from **Figure 3**).

Blood samples drawn 6 days after the Hib-MenC-TT booster were used to compare primed children (groups 1, 2, and 4) and un-primed (control) children (Figure 4). A threshold of 2.5 MenC memory B-cells/million PBMCs was picked by visually inspecting the data and drawing a line through the point that would best separate results from the primed compared to the un-primed children. Use of this “threshold” as a test to detect primed children gives a sensitivity of 0.86 and specificity of 0.89.

### Kinetics of memory B-cell generation

**Figure 5:**
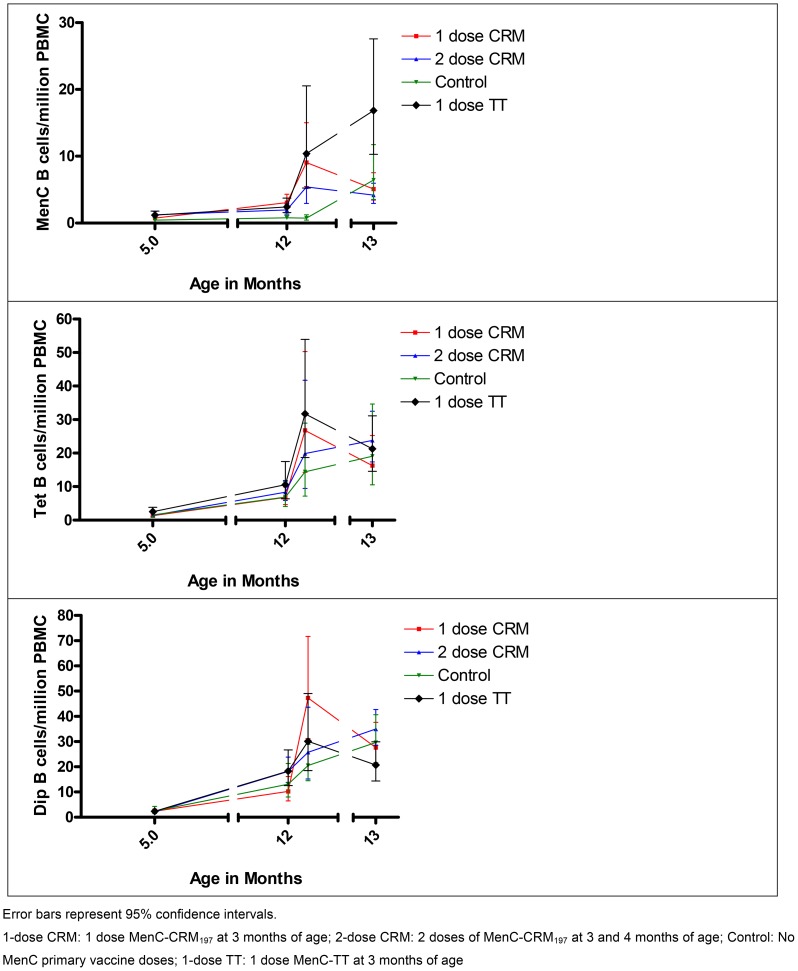
Kinetics of the number of antigen-specific memory B-cells detected in the peripheral blood of infants after immunisation with different schedules of MenC conjugate vaccines, at each time-point following primary and booster vaccines, based on geometric mean concentrations for each study group at each visit.

The kinetics of MenC memory B-cell generation over time for each group is shown in figure 5 based on geometric mean concentrations calculated for the number of MenC-specific memory B-cells detected at each time-point for each group. The kinetics of memory B-cell production for the control antigens, diphtheria (Dip) and tetanus (Tet) are also included. The patterns for each group appear to be antigen-specific.

Using linear mixed models of the log_10_ transformed data, a statistically significant rise was seen in the number of memory B-cells between 5 months (1 month after primary vaccines) and 12 months of age (prior to booster vaccines) across all antigens tested with the exception of MenC-specific memory B-cells in the control children. P-values for the differences between the 5-month and 12-month samples (when compared with a difference of 0) are reported in Table S3 in file S1. To exclude variability in the assay over time, MenC-specific memory B-cell responses for all children were plotted according to calendar month of blood sampling for the 5-month and 12-month samples. No change in the pattern of results was seen over time (results not included).

## Discussion

This is the first study to evaluate serogroup C meningococcal memory B-cell responses following different priming schedules in infants with different conjugate vaccines, and has surprisingly found that priming does not produce a significant improvement in the memory response to a booster dose of vaccine 7 months later in comparison to unprimed controls. Furthermore, no evidence was seen in this study for a relationship between the number of priming doses of MenC-CRM_197_, and the generation of MenC-specific memory B-cells at any time-point following primary or booster immunisations in infants. These findings are in contrast to previously published studies which examined immunological memory after immunisation using antibody responsiveness as a surrogate and found that antibody responses following a booster were higher in those who had received fewer priming vaccine doses. Borrow *et al* [11] showed that infants primed with 1 dose of MenC-TT in the first year of life, mounted a greater antibody response to a polysaccharide challenge at 12 months of age, compared to priming with either 2 or 3 doses of MenC-TT. A more recent study has shown that antibody levels following a MenC-TT booster at 12 months of age are higher in children who received 1 dose of the same vaccine at 4 months of age, compared to children who received 2 doses at 2 and 4 months [10]. Similar results were demonstrated in a study comparing 1, 2 or 3 doses of pneumococcal conjugate vaccine in infancy, followed by a dose of pneumococcal polysaccharide vaccine at 12 months of age [17]. Children who had received a single dose of priming vaccine mounted a greater serotype-specific antibody response following polysaccharide challenge than children who had received 2 or 3 priming doses. These previous studies imply that limiting the number of “priming” doses of vaccine may favour memory formation, at the expense of initial antibody response, potentially providing longer term protection. The results of the immunogenicity component of the current study confirmed these previous findings, demonstrating significantly greater MenC SBA titres following Hib-MenC-TT booster in children previously primed with a single dose, compared to two priming doses of MenC-CRM_197_ [18]. However, with very small numbers of memory B-cells detected before booster vaccination, the current study may have lacked power to detect a difference between 1- and 2-dose priming with MenC-CRM_197_ conjugate vaccine with respect to memory B-cell responses.

It is not clear what signals are required to determine memory B-cell production following activation of naïve B-cells. It has been suggested that a population of naïve B-cells are intrinsically able to commit to any fate based on stochastic factors influenced by a large number of external and intrinsic signals [19], [20]. Some of these signals may favour differentiation into plasma cells over memory cells, or vice versa, and may include the type or amount of antigen used for immunization [21]. In the current study, the number of MenC-specific memory B-cells detected by the ELISpot assay was shown to be related to the type of vaccine used for priming and boosting. Whether these differences are related to the carrier proteins used in these vaccines, or other compositional differences is uncertain. Children primed with MenC-TT conjugate vaccine generated more memory B-cells following a Hib-MenC-TT booster than children previously primed with MenC-CRM_197_, suggesting that the carrier protein used for priming and boosting may have an important role in determining the polysaccharide-specific response to conjugate vaccines. These data may provide indirect evidence to support the recent finding from murine studies that an association of peptides from the carrier protein, along with oligosaccharides from the sugar component of conjugate vaccines, are presented together to T-cells on major histocompatibility complex (MHC)-II receptors, challenging the traditional dogma about the mechanism of immune induction by conjugate vaccines [22]. The structure of the MenC-CRM_197_ polysaccharide-conjugate vaccine may interfere with antigen processing and/or presentation on MHC-II molecules following immunisation, whereas that of the MenC-TT conjugate vaccine may promote optimal presentation of carbohydrate epitopes recognised by T-cells. If this mechanism is verified in humans, it has important implications for new generation conjugate vaccine development.

The differences between vaccines in induction of MenC-specific memory B-cells may explain the differential antibody response noted following primary and booster vaccines when children were primed with either a MenC-CRM_197_ vaccine or Hib-MenC-TT in the first year of life and given a Hib-MenC-TT booster as toddlers [8], [9]. In these earlier studies, children in the MenC-CRM_197_ primed groups had higher SBA titres one month after primary vaccines, but lower SBA titres one month after the Hib-MenC-TT booster, than children in the Hib-MenC-TT primed groups. Similarly, children primed with MenC-TT have been shown to mount a greater SBA response following either MenC-TT or MenC-CRM_197_ booster in the second year of life, than children primed with MenC-CRM_197_ [7]. These observations may be related to a greater number of MenC memory B-cells generated by Hib-MenC-TT and MenC-TT than by MenC-CRM_197_ primary immunisations. Although in the current study no statistically significant difference was seen in the number of memory B-cells at 5 months of age in children who had received either MenC-TT or MenC-CRM_197_ at 3 months of age, there was a trend towards higher numbers in the MenC-TT group (data not presented).

In the absence of immunisation programmes, adolescents and young adults have a high incidence of invasive meningococcal disease and high case fatality rate (10–14%) compared to younger children and to adults, and are also the drivers for transmission of the organism through high nasopharyngeal carriage rates (10–35%) [23]. The only validated correlate of protection against invasive MenC disease is an SBA titre ≥1∶8 using rabbit complement or ≥1∶4 using human complement [1], [2], [3]. Thus persistence of bactericidal antibody beyond childhood is critical for both direct protection of individuals through the “high risk” period of adolescence, and for maintenance of herd immunity. The level of MenC-specific memory B-cells produced after priming vaccines has been shown to correlate with the persistence of antibody and memory B-cells at one year of age, and with post-booster antibody and memory B-cell levels [12]. In addition, the persistence of MenC bactericidal antibody through early childhood is greater in children who mount a greater response to the toddler booster dose of vaccine [24]. Thus, vaccines which induce more memory B-cells following primary and booster immunisations may be associated with greater persistence of bactericidal antibody throughout childhood and adolescence and offer a practical advantage for infant immunisation schedules.

In this study, a rise in antigen-specific memory B-cells was seen following primary immunisations until booster vaccination at 12 months of age for all antigens tested. Variability in the assay over time was excluded and for MenC, this finding was consistent with an increase in the proportion of MenC-specific memory B-cells out of the total pool of IgG positive memory B-cells from 0.02% at 5 months to 0.04% at 12 months of age, suggesting that this is an antigen-specific increase, rather than a non-specific effect of immune maturation in infants. Furthermore, the absence of a rise in the number of MenC-specific memory B-cells in unprimed children suggests that this a true effect of priming on memory B-cell production. However, the rise in antigen-specific memory B-cells in this study is in contradiction to the findings of Blanchard *et al* who noted a decline in MenC memory B-cells between 5 and 12 months of age following 3 doses of MenC-CRM_197_ at 2, 3 and 4 months of age [12]. The main difference between these two studies is in the number of memory B-cells detected at 5 months of age: median 0.1–0.8 per million PBMCs in children primed with MenC-CRM_197_ in the current study versus 11 per million PBMCs in the Blanchard *et al* trial. This may be related to the number of primary doses of vaccine administered (1 or 2 in the current study versus 3 in the previous trial). Furthermore, Blanchard *et al* used different concentrations of SAC and CpG which may have led to greater B-cell stimulation and higher spot counts in general. The differences are unlikely to be related to changes in carriage rates of MenC in the UK between 2005 and 2011/2012, since even prior to the introduction of MenC conjugate vaccines only 0.45% of adolescents were colonised with MenC, and this had reduced to 0.15% by 2000 [4], with carriage rates in infants being virtually zero.

The formation of new memory B-cells for several months after immunisation suggests that this may either be occurring independent of the germinal centre (GC), or alternatively, in these infants, the GC reaction may continue for longer than is traditionally understood. The memory B-cell population in humans and higher vertebrates is known to be heterogeneous [21]. In mice, antigen-specific B-cells with a memory phenotype have been demonstrated early in a GC reaction, with evidence of stable numbers or continued output for at least 5 weeks [25]. In addition, studies in mice indicate that GC-independent memory B-cells can be formed [26], [27] and subsets of memory B-cells continue to proliferate in GC-like structures in splenic B-cell follicles for up to 8 months after immunization [28]. In humans, memory B-cells have been shown to continue to proliferate and differentiate into plasma cells in lymphoid tissues for several months after immunisation in response to antigen persisting on follicular dendritic cells [29]. In line with the mouse models described above, our results suggest that memory B-cells can continue to be generated for months beyond the original antigen stimulation.

In the context of waning bactericidal antibody described in the first year of life following infant immunizations [8], [30], an increase in the number of MenC-specific memory B-cells between 5 and 12 months of age suggests that after primary MenC vaccines, memory B-cells continue to be generated for several months, but there is a decline in the proportion that differentiate into plasma cells. Despite waning of bactericidal antibody levels, antibody avidity has also been shown to increase following primary immunisations with MenC and pneumococcal conjugate vaccines in infants, until 12 months of age [11], [31]. One explanation for the discordance in antibody levels (waning over time) and antibody avidity and memory B-cell numbers (increasing over time) may be that separate B-cell precursors are responsible for the primary antibody-producing and memory B-cell populations [32]. It should be noted however, that as no blood samples were drawn in this study between 5 and 12 months of age, we cannot exclude a higher peak in memory B-cell numbers at an earlier time-point after 5 months, with a decline until 12 months of age.

Primary and secondary GC reactions have been shown to be qualitatively very similar [33] although there are several quantitative differences, including the speed with which they develop. During a secondary response, IgG memory B-cells appear in the circulation more rapidly, and in adolescents receiving a booster dose of MenC conjugate vaccine memory B-cells were detected by day 6 [34]. For a tetanus booster in adults an increase in memory B-cells was not seen by day 4 but by day 12 there was a significant rise [14]. In infants, after the third dose of vaccine antigen-specific memory B-cells were readily detectable at all time-points from day 4 to 30 [13]. Most clinical vaccine trials compare pre- and post- booster antibody levels to detect an anamnestic response; often one month apart and based on a polysaccharide vaccine “challenge”. In the current study, detection of at least 2.5 MenC memory B-cells/million PBMCs in the peripheral blood, 6 days after a booster dose of Hib-MenC-TT was able to differentiate MenC-primed from un-primed children with a sensitivity and specificity of >85%. This could potentially be used as a novel way to test the ability of MenC conjugate vaccines to induce immune memory in infants and young children with the following advantages: 1) results are available from a single blood test, 2) earlier time-point to achieve results, 3) avoids the need for administration of a polysaccharide vaccine as a ‘challenge’ dose in this population where there are concerns about possible induction of immunological hypo-responsiveness [35]. This finding needs to be validated by testing in blinded fashion in other cohorts of children, as well as with other conjugate vaccines; however this study provides a “proof of principle” for using the ELISpot assay for detecting antigen-specific memory B-cells on the 6 -day post-booster blood sample to identify previously “primed” individuals.

## Conclusions

MenC-specific memory B-cell production may be more dependent on the type of vaccine used for primary immunisation of infants, and perhaps the matching of carrier proteins, than the number of doses administered. Although the mechanistic differences between MenC-CRM_197_ and MenC-TT priming are unclear, it is possible that structural differences in these vaccines may underlie differential interactions with B- and T-cell populations and thus different effects on various memory B-cell subsets. The differences between these vaccines in induction of MenC-specific memory B-cells may explain the differential antibody response noted following primary and post Hib-MenC-TT booster immunisations. The MenC-TT/Hib-MenC-TT combination of vaccines may offer a practical advantage for infant immunisation schedules in terms of long-term persistence of antibody. Irrespective of the priming vaccine received, MenC primed children can be separated from unprimed children with a sensitivity and specificity of >85% by the detection of at least 2.5 MenC memory B-cells/million PBMCs in the peripheral blood, 6 days after a Hib-MenC-TT booster vaccine.

## Supporting Information

Checklist S1 **CONSORT Checklist.**
(DOC)

Protocol S1 **Clinical Study Protocol.**
(DOC)

File S1 **Supplemental data tables: Table S1**: Comparison of the log_10_ transformed number of MenC-specific memory B-cells detected in the peripheral blood for each study group against the control group at visits 4, 5 and 6 (5 months, 12 months and 12 months +6 days). **Table S2**: Differences in log_10_ transformed number of MenC-specific memory B-cells detected in the peripheral blood between 13-month and 12-month samples for each study group. **Table S3**: Differences in the log_10_ transformed number of antigen-specific memory B-cells detected in the peripheral blood between 5 months and 12 months for each study group.
(DOCX)
