# Supplementary material for: Evaluation of the Induction of Immune Memory following Infant Immunisation with Serogroup C Neisseria meningitidis Conjugate Vaccines – Exploratory Analyses within a Randomised Controlled Trial
Source: PLoS One. 2014 Jul 14;9(7):e101672. doi: 10.1371/journal.pone.0101672 (PMC4096514; doi:10.1371/journal.pone.0101672)
Supplement: Protocol S1 — Clinical Study Protocol. (DOC) [file pone.0101672.s002.doc]

Study Title: **An open label randomised controlled study to evaluate the induction of immune memory following infant vaccination with a glyco-conjugate *Neisseria meningitidis* serogroup C vaccine and to assess the immune response to the concurrent infant routine immunisations administered in consistent versus alternating limbs**

Internal Reference No: OVG 2008/6

Ethics Ref: 10/H0604/7

Eudract Number: 2009-016579-31

Date and Version No: 29.09.2012 Version 11

| Chief Investigator: | Professor Andrew J Pollard |
| --- | --- |
| Investigators: | Dr Matthew Snape, Dr David Pace, Dr Ameneh Khatami, Dr Andrew Marshall, Professor Adam Finn, Dr Saul Faust, Dr Paul Heath |
| Sponsor: | University of Oxford |
| Funder: | NIHR Oxford Biomedical Research Centre |
| Signature of Chief Investigator: |  |

Confidentiality Statement

This document contains confidential information that must not be disclosed to anyone other than the Sponsor, the Investigator Team, host NHS Trust (s), regulatory authorities, and members of the Research Ethics Committee.

TABLE OF CONTENTS

[1. AMENDMENT HISTORY 4](#__RefHeading___Toc266369893)

[2. SYNOPSIS 6](#__RefHeading___Toc266369894)

[3. ABBREVIATIONS 13](#__RefHeading___Toc266369895)

[4. BACKGROUND AND RATIONALE 15](#__RefHeading___Toc266369896)

[5. OBJECTIVES 22](#__RefHeading___Toc266369897)

[5.1 Primary Objective 22](#__RefHeading___Toc266369898)

[5.2 Secondary Objectives 22](#__RefHeading___Toc266369900)

[6.2 Primary and Secondary Endpoints/Outcome Measures 26](#__RefHeading___Toc266369902)

[6.3.1 Overall Description of Trial Participants 30](#__RefHeading___Toc266369903)

[6.3.2 Inclusion Criteria 30](#__RefHeading___Toc266369904)

[6.3.4 Elimination criteria during the study 31](#__RefHeading___Toc266369905)

[6.5 Definition of End of Trial 38](#__RefHeading___Toc266369906)

[6.6 Discontinuation/ Withdrawal of Participants from Study Treatment 39](#__RefHeading___Toc266369907)

[6.7 Source Data 39](#__RefHeading___Toc266369908)

[7. TREATMENT OF TRIAL PARTICIPANTS 40](#__RefHeading___Toc266369909)

[7.1 Description of Study Treatment 40](#__RefHeading___Toc266369910)

[7.1.1 Dosage and administration 40](#__RefHeading___Toc266369911)

[7.3 Compliance with Study Treatment 41](#__RefHeading___Toc266369912)

[7.4 Accountability of the Study Treatment 41](#__RefHeading___Toc266369913)

[7.5 Concomitant Medication 41](#__RefHeading___Toc266369914)

[8.1 Definitions 42](#__RefHeading___Toc266369915)

[8.2 Reporting Procedures for All Adverse Events 44](#__RefHeading___Toc266369916)

[Reporting Procedures for Serious Adverse Events 44](#__RefHeading___Toc266369917)

[9 STATISTICS 45](#__RefHeading___Toc266369918)

[9.1 Description of Statistical Methods 45](#__RefHeading___Toc266369920)

[9.2 The Number of Participants 55](#__RefHeading___Toc266369921)

[9.3 Hypothesis Test 57](#__RefHeading___Toc266369922)

[9.4 Criteria for the Termination of the Trial. 57](#__RefHeading___Toc266369923)

[9.5 Procedure for Accounting for Missing, Unused, and Spurious Data. 58](#__RefHeading___Toc266369924)

[9.6 Procedures for Reporting any Deviation(s) from the Original Statistical Plan 58](#__RefHeading___Toc266369925)

[9.7 Inclusion in Analysis 58](#__RefHeading___Toc266369926)

[10 Direct Access to Source Data/Documents 58](#__RefHeading___Toc266369927)

[11 Quality Control and Quality Assurance Procedures 58](#__RefHeading___Toc266369928)

[12 Ethics 59](#__RefHeading___Toc266369929)

[12.1 Declaration of Helsinki 59](#__RefHeading___Toc266369930)

[12.2 ICH Guidelines for Good Clinical Practice 59](#__RefHeading___Toc266369931)

[12.3 Approvals 59](#__RefHeading___Toc266369932)

[12.4 Participant Confidentiality 59](#__RefHeading___Toc266369933)

[13 Data Handling and Record Keeping 60](#__RefHeading___Toc266369934)

[14 Financing and Insurance 60](#__RefHeading___Toc266369935)

[15 Publication Policy 60](#__RefHeading___Toc266369936)

[16 Storage and handling of samples 60](#__RefHeading___Toc266369937)

[17 REFERENCES 62](#__RefHeading___Toc266369938)

[18 APPendix a: study flow CharT 66](#__RefHeading___Toc266369939)

[19 APPENDIX B: SCHEDULE OF PROCEDURES 68](#__RefHeading___Toc266369940)

[20 Appendix C: Intervals between Visits 69](#__RefHeading___Toc266369941)

# AMENDMENT HISTORY

| **Amendment No.** | **Protocol Version No.** | **Date issued** | **Author(s) of changes** | **Details of Changes made** |
| --- | --- | --- | --- | --- |
| 1 | 2 | 05.02.10 | A Khatami | PCV13 to replace PCV7  Menjugate Kit to replace NeisVac-C  Delay criteria for vaccination |
| 2 | 3 | 22.04.10 | A Khatami | Addition of new study group  Addition of 24 month persistence sample |
| 3 | 4 | 12.05.10 | D Pace  A Khatami | Addition of new study sites, and site specific procedures.  Clarification of statistical analyses, randomisation and blinding.  Clarification of sample handling procedures.  Clarification of concomitant vaccines permitted in the trial  Addition of trial steering committee  Correction of typographical error in Appendix C.  Removal of repeated secondary endpoints.  Addition of new objective and endpoint.  Clarification of Group numbers |
| 4 | 5 | 26.11.10 | A Khatami | Clarification of recruitment procedures  Clarification of adverse event reporting procedures |
| 5 | 6 | 23.02.11 | A Khatami | Clarification of recruitment procedures  Inclusion of Varicella Vaccine to list of non-study vaccines permitted. |
| 6 | 7 | 03.06.11 | A Khatami | Inclusion of Hep A Vaccine to list of non-study vaccines permitted. |
| 7 | 8 | 06.10.11 | A Khatami | Inclusion of interim analysis |
| 8 | 9 | 03.11.11 | A Khatami and D Pace | Clarification of statistical analyses and inclusion of a second attempt to blood sampling if first is unsuccessful |
|  | 9 | 13.12.11 | A Khatami | Correction of typing errors on page 45 and 57 |
| 9 | 10 | 30.05.2012 | A Tajar | Re-wording of hypothesis tests |
| 10 | 11 | 29.05.2012 | A Khatami | Clarification of V5 timelines and elimination criteria |

# SYNOPSIS

| **Study Title** | **An open label randomised controlled study to evaluate the induction of immune memory following infant vaccination with a glyco-conjugate *Neisseria meningitidis* serogroup C vaccine and to assess the immune response to the concurrent infant routine immunisations administered in consistent versus alternating limbs** |
| --- | --- |
| **Internal ref. no.** | OVG 2008/6 |
| **Clinical Phase** | Phase IV |
| **Trial Design** | Open label randomised controlled trial |
| **Trial Participants** | 6 - 12 week old infants |
| **Planned Sample Size** | 498 participants |
| **Follow-up duration** | 24 months |
| **Planned Trial Period** | 2010 – 2013 |
| **Primary Objective** | The primary objective of this study is to demonstrate non-inferiority of the geometric mean titres (GMTs) of meningococcal serogroup C (MenC) specific serum bactericidal antibodies, using rabbit complement (rSBA), 1 month after a 12 month dose of Hib-MenC vaccine in children receiving a single dose of MenC-CRM_197_ vaccine at 3 months of age (single dose priming, Group 1) compared with those receiving 2 doses at 3 and 4 months of age (2 dose priming, Group 2). Non inferiority of the MenC serum bactericidal antibody geometric mean titres (SBA GMTs) would imply that the reduced schedule of MenC immunisation would be a more cost effective method of providing sustained immunity against MenC disease through childhood. |
| **Secondary Objectives** | Reduced dose MenC component:   1. To assess whether MenC SBA GMTs measured 1 month after a 12 month dose of Hib-MenC are higher in children previously receiving single dose MenC-CRM_197_ vaccine priming at 3 months of age (Group 1) than in those receiving no priming doses of MenC vaccine (Group 3), demonstrating whether single dose MenC priming offers any advantage over no priming in terms of antibody levels at 13 months, and whether a single infant dose of MenC vaccine induces immune memory. 2. In an exploratory analysis, to compare the MenC SBA GMTs at day 6 after the 12 month dose of vaccine in a subset of participants (64 participants from the Single dose MenC-CRM_197_ [Group 1], Two dose MenC [Group 2] and Single Dose MenC-TT [Group 4] groups and all 64 in the control group [Group 3]: n=256) from all four groups, as it has been proposed that assessment of specific antibody levels at this earlier time point will more effectively discriminate between 'primed' and 'unprimed' immune responses to vaccines. 3. To assess the MenC SBA GMTs 2 months after a dose of MenC-CRM_197_ vaccine at 3 months of age (Group 1) compared to the MenC SBA GMTs taken 1 month after a course of MenC-CRM_197_ vaccine at 3 and 4 months of age (Group 2), and to a control group (Group 3) receiving no infant MenC immunisation who would be sampled at 5 months of age. 4. To assess MenC SBA GMTs 2 months after a dose of a MenC-TT vaccine at 3 months of age (Single Dose MenC-TT Group, Group 4) compared to a single dose of a MenC-CRM_197_ conjugate vaccine (Single Dose MenC-CRM_197_ Group, Group 1), on a blood sample taken at 5 months of age. 5. To assess MenC SBA GMTs measured 12 months after a 12 month dose of Hib-MenC in all groups receiving priming vaccines with MenC-CRM_197_, or no priming (Groups 1, 2 and 3), to determine whether 2 dose MenC priming offers any advantage over single dose MenC priming or no priming in terms of antibody levels at 24 months. 6. To assess MenC SBA GMTs 12 months after a 12 month dose of Hib-MenC in children previously receiving a single dose of a MenC-TT vaccine at 3 months of age (Group 4) compared to a single dose of a MenC-CRM_197_ conjugate vaccine (Group 1). 7. It is intended to measure the numbers of MenC specific memory B cells in the blood at 5 months, 12 months, 6 days following the 12 month booster dose, 13 months and 24 months of age, on a subset of participants. Specific memory B cells against diphtheria and tetanus will also be measured and used as a control. As many participants as possible would be included in this subset but the number would be determined by the practicalities of getting the blood to the laboratory in time (before midday) for processing. 8. To assess the local and systemic adverse reactions experienced by participants in the four different study groups after immunisation with each dose of the MenC-CRM_197_, MenC-TT and Hib-MenC vaccines.   Alternating limb component   1. To compare the *S. pneumonia*e IgG GMCs and percentage of infants with serum concentration of *S. pneumonia*e specific IgG ≥0.35 μg/ml for all 13 PCV13 serotypes at 5, 12, 13 and 24 months of age in children receiving PCV13 in a consistent limb (subgroups ‘a’) vs those receiving this in alternating limbs (subgroups ‘b’). 2. To compare serotype specific pneumococcal B cell phenotype in a subset of participants at 5, 12, 13 and 24 months of age in children receiving PCV13 in a consistent limb (sub groups ‘a’) vs those receiving this in alternating limbs (subgroups ‘b’). 3. To compare the anti-PRP IgG GMCs and percentage of infants with serum concentration of anti-PRP IgG ≥0.15μg/ml and ≥1.0 μg/ml and at 5, 12 13 and 24 months of age in children receiving DTaP-IPV-Hib in a consistent limb (subgroups ‘a’) vs those receiving this in alternating limbs (subgroups ‘b’). 4. To compare the anti-tetanus toxoid GMCs and percentage of infants with anti-tetanus toxoid >0.1 IU/ml at 5, 12, 13 and 24 months of age in participants receiving DTaP-IPV-Hib in a consistent limb (sub groups ‘a’) vs those receiving this in alternating limbs (subgroups ‘b’). 5. To compare the local or systemic vaccine reactions in subgroups a and b at each vaccination time point. |
| **Primary Endpoint** | The difference in the MenC rSBA GMTs between the participants primed with two doses of MenC-CRM_197_ (Two Dose MenC Group, Group 2) and with one dose of MenC-CRM_197_ (Single Dose MenC-CRM_197_ Group, Group 1), one month following the Hib-MenC booster dose at 12 months. |
| **Secondary Endpoints** | After administration of the Hib-MenC booster dose at 12 months of age the following comparisons would be performed in order to assess differences between the:  1) MenC rSBA GMTs between the Two Dose MenC Group and the Control group at 6 and 28 days and 12 months later (Group 2 vs Group 3)  2) MenC rSBA GMTs between the Single dose MenC-CRM_197_ group and the Control group at 6 and 28 days and 12 months later (Group 1 vs Group 3)  3) MenC rSBA GMTs between the Single Dose MenC-TT group and the Control group at 6 and 28 days and 12 months later (Group 4 vs Group 3)  4) MenC rSBA GMTs between the Single Dose MenC-CRM_197_ group and the Single Dose MenC-TT group at 6 and 28 days and 12 months later (Group 1 vs Group 4)  5) Percentage of participants with MenC rSBA ≥1:8 and ≥1:128 between the Two Dose MenC group and the Control group at 6 and 28 days and 12 months later (Group 2 vs Group 3)  6) Percentage of participants with MenC rSBA ≥1:8 and ≥1:128 between the Single dose MenC-CRM_197_ group and the Control group, at 6 and 28 days and 12 months later (Group 1 vs Group 3)  7) Percentage of participants with MenC rSBA ≥1:8 and ≥1:128 between the Two Dose MenC Group and the Single Dose MenC-CRM_197_ group, at 6 and 28 days and 12 months later (Group 2 vs Group 1)  8) Percentage of participants with MenC rSBA ≥1:8 and ≥1:128 between the Single Dose MenC-TT group and the Control group at 6 and 28 days and 12 months later (Group 4 vs Group 3)  9) Percentage of participants with MenC rSBA ≥1:8 and ≥1:128 between the Single Dose MenC-CRM_197_ group and the Single Dose MenC-TT group at 6 and 28 days and 12 months later (Group 1 vs Group 4)  10) Percentage of participants with MenC rSBA GMTs >1000 between the Two Dose MenC Group and the Control Group, at 6 and 28 days and 12 months later (Group 2 vs Group 3)  11) Percentage of participants with MenC rSBA GMTs >1000 between the Single Dose MenC-CRM_197_ Group and the Control Group, at 6 and 28 days and 12 months later (Group 1 vs Group 3)  12) Percentage of participants with MenC rSBA GMTs >1000 between the Two Dose MenC Group and the Single Dose MenC-CRM_197_ Group, at 6 and 28 days and 12 months later (Group 2 vs Group 1)  13) Percentage of participants with MenC rSBA GMTs >1000 between the Single Dose MenC-TT group and the Control group at 6 and 28 days and 12 months later (Group 4 vs Group 3)  14) Percentage of participants with MenC GMTs >1000 between the Single Dose MenC-CRM_197_ group and the Single Dose MenC-TT group at 6 and 28 days and 12 months later (Group 1 vs Group 4)  After administration of the last MenC-CRM_197_ dose at 4 months of age the following comparisons would be performed to assess differences between:  15) MenC rSBA GMTs between the Two Dose MenC Group vs Single Dose MenC-CRM_197_ Group (Group 2 vs Group 1), Two Dose MenC Group vs Control Group (Group 2 vs Group 3), Single Dose MenC-CRM_197_ vs Control Group (Group 1 vs Group 3), Single Dose MenC-TT vs Control group (Group 4 vs Group 3), and Single Dose MenC-CRM_197_ Group vs Single Dose MenC-TT group (Group 1 vs Group 4) at 5 and 12 months of age  16) Percentage of participants with MenC rSBA ≥1:8 between the Two Dose MenC Group vs Single Dose MenC-CRM_197_ Group (Group 2 vs Group 1), Two Dose MenC Group vs Control Group (Group 2 vs Group 3), Single Dose MenC-CRM_197_ vs Control Group (Group 1 vs Group 3), Single Dose MenC-TT vs Control Group (Group 4 vs Group 3), Single Dose MenC-CRM_197_ Group vs Single Dose MenC-TT Group (Group 1 vs Group 4) at 5 and 12 months of age  17) Percentage of participants with MenC rSBA ≥1:128 between the Two Dose MenC Group vs Single Dose MenC-CRM_197_ Group (Group 2 vs Group 1), Two Dose MenC Group vs Control Group (Group 2 vs Group 3), Single Dose MenC-CRM_197_ vs Control Group (Group 1 vs Group 3), Single Dose MenC-TT vs Control Group (Group 4 vs Group 3), Single Dose MenC-CRM_197_ Group vs Single Dose MenC-TT group (Group 1 vs Group 4) at 5 and 12 months of age  18) Number of MenC memory B cells at 5 months, 12 months, 12 months+6 days, 13 months and 24 months (on a subset of participants) between the Two Dose MenC Group vs Single Dose MenC-CRM_197_ Group (Group 2 vs Group 1), Two Dose MenC Group vs Control Group (Group 2 vs Group 3), Single Dose MenC-CRM_197_ vs Control Group (Group 1 vs Group 3), Single Dose MenC-TT vs Control Group (Group 4 vs Group 3), Single Dose MenC-CRM_197_ Group vs Single Dose MenC-TT (Group 1 vs Group 4).  Alternating limb component  After administration of the DTaP-IPV-Hib and PCV-13 the following comparisons would be performed to assess differences between the:   1. Anti-*S. pneumoniae* IgG GMCs or percentage of infants with serum concentration of *S. pneumonia*e specific IgG ≥0.35 μg/ml for each of the 13 serotypes at 5, 12, 13 and 24 months of age in children receiving PCV13 in a consistent limb (sub groups ‘a’) vs those receiving this in alternating limbs (subgroups ‘b’). 2. Serotype specific pneumococcal B cell phenotype in a subset of participants at 5, 12, 13 and 24 months of age in children receiving PCV13 in a consistent limb (sub groups ‘a’) vs those receiving this in alternating limbs (subgroups ‘b’). 3. Anti-PRP IgG GMCs or percentage of infants with serum concentration of anti-PRP IgG ≥0.15μg/ml and ≥1.0 μg/ml at 5, 12, 13 and 24 months of age in children receiving DTaP-IPV-Hib in a consistent limb (sub groups ‘a’) vs those receiving this in alternating limbs (subgroups ‘b’). 4. Anti-tetanus toxoid GMCs or percentage of participants with anti-tetanus toxoid >0.1IU/ml at 5, 12, 13 and 24 months of age in children receiving DTaP-IPV-Hib in a consistent limb (sub groups ‘a’) vs those receiving this in alternating limbs (subgroups ‘b’). |
| **Investigational Medicinal Products** | All vaccines to be used are licensed.  MenC-CRM_197_ vaccine (*Menjugate,* Novartis Vaccines and Diagnostics)  MenC-TT vaccine (*NeisVac-C*, Baxter Healthcare)  DTaP-IPV-Hib (*Pediacel*, Sanofi Pasteur MSD )  Hib-MenC-TT (*Menitorix*, GlaxoSmithKline Biologicals)  PCV13 (*Prevenar-13*, Wyeth Vaccines) |

# ABBREVIATIONS

AE Adverse event

AR Adverse reaction

ATP According to protocol

CI Chief Investigator

CRF Case Report Form

CRO Contract Research Organisation

CT Clinical Trials

CTA Clinical Trials Authorisation

CTRG Clinical Trials & Research Governance, University of Oxford

DTaP-IPV-Hib Pediacel (Sanofi Pasteur, MSD)

EC Ethics Committee (see REC)

GCP Good Clinical Practice

Hib-MenC Menitorix (GlaxoSmithKline Biologicals)

IB Investigators Brochure

ICF Informed Consent Form

ICH International Conference of Harmonisation

IEC Independent Ethics Committee

IMP Investigational Medicinal Products

IRB Independent Review Board

ITT Intention to treat

MenC Serogroup C meningococcal

MMR Measles, mumps and rubella vaccine

MHRA Medicines and Healthcare products Regulatory Agency

NHS National Health Service

NRES National Research Ethics Service (previously known as COREC)

OVG Oxford Vaccine Group

PCV13 Prevenar-13 (Wyeth Vaccines)

PI Principal Investigator

PIL Participant/ Patient Information Leaflet

R&D NHS Trust R&D Department

REC Research Ethics Committee

SAE Serious Adverse Event

SAR Serious Adverse Reaction

SIL Subject Information Leaflet (see PIL)

SmPC/SPC Summary of Products Characteristics

SOP Standard Operating Procedure

SUSAR Suspected Unexpected Serious Adverse Reactions

TMF Trial Master File

TSG Oxford Radcliffe Hospitals Trust / University of Oxford Trials Safety

Group

# BACKGROUND AND RATIONALE

**BACKGROUND:**

*Neisseria meningitidis* is a major cause of meningitis and septicaemia globally with the greatest burden of endemic disease occurring in infants, children below 4 years of age and in adolescents^1,2^. However, during epidemics older children and adults are also affected^3^. *N. meningitidis* serogroups A, B, C, Y and W-135 cause the majority of invasive meningococcal disease worldwide with serogroups B and C accounting for more than 90% of cases in Europe and the US^1,2^. Because of the fulminant nature of the disease the overall case fatality rate remains at 4-10%, in spite of the prompt initiation of effective antibiotics and advances in intensive care^4,5^. 11-19% of survivors often sustain permanent disabilities, including neurological and intellectual impairment, amputations and hearing loss^6^. Vaccination is the only rational strategy for prevention of meningococcal disease.

In the 1990s an increase in the number of cases caused by the ST11 hyperinvasive clone of serogroup C was observed in Europe and the US^7^. This led to the formulation and development of three protein-polysaccharide conjugate MenC vaccines; two CRM197 conjugates: *Menjugate* (Novartis Vaccines and Diagnostics, Siena, Italy), and *Meningitec* (Wyeth Vaccines, Pearl River, New York) and one utilising tetanus toxoid as a carrier protein: *Neisvac-C* (Baxter Vaccines Beltsville, MD). Pre-licensure clinical trials showed that in contrast to plain polysaccharide meningococcal vaccines, these MenC conjugate vaccines resulted in the production of bactericidal antibodies from infancy, due to their ability to recruit T-cell help and the subsequent stimulation of immune memory^8^. Because of the rise in MenC disease and the availability of safety and immunogenicity data, these three MenC vaccines were first licensed in the UK in 1999 and used for a mass immunisation campaign directed against children and adolescents, despite the lack of formal efficacy data. These vaccines are used interchangeably in the UK immunisation schedule and are given at 3 and 4 months of age, with a combined Hib-MenC vaccine given as a booster dose at 12 months of age (this schedule was introduced in 2006 but from 1999-2006 a schedule of 3 doses at 2, 3 and 4 months of age without a booster was used). Other countries adopting routine immunisation against MenC disease have used alternative immunisation schedules, with many, such as Australia and The Netherlands, opting for a single dose of MenC at 12 months of age.^7^ The MenC vaccine has been introduced in Malta in 2009 and although not yet part of the national immunisation schedule, because of budgetary restrictions, is available privately where it is administered according to the UK schedule.

The administration of conjugate vaccines during clinical trials is usually standardised in a way that sequential doses of the study vaccine are administered in the same limb. Such practice may theoretically result in a better immune response due to stimulation of a greater number of memory B and T cells resident in draining lymph nodes previously primed by the same vaccine antigens^9,10^. However, after licensure sequential doses of these vaccines are not usually administered as such and in many instances are administered in alternating limbs.

**RATIONALE:**

The purpose of this study is to evaluate the most appropriate immunisation schedule against MenC disease for UK children in the current era where the disease is under control. In Malta the MenC vaccine is not yet part of the national immunisation programme but has become available privately since late 2009. Malta has the highest incidence rate of invasive meningococcal disease (IMD) in Europe (crude incidence rate of 8.1 per 100,000 population) and epidemiological data from 1994-2006 have shown that although the majority are caused by MenB, 16% of all cases of microbiologically confirmed invasive meningococcal disease are caused by MenC^11,12^. In Malta children from 1-14 years of age suffer the majority of the total burden of IMD. Due to the lack of molecular techniques in identifying the meningococcus, 52% of cases of IMD which fit the clinical diagnostic criteria are unconfirmed which would suggest that the disease burden of MenC might be greater^11^. The introduction of routine MenC vaccination could have a modest reduction of IMD in Malta. This study would provide essential information on the most beneficial and cost-effective MenC schedule and will be crucial in determining how the MenC vaccine would best fit in the current national immunisation programme, when introduced.

This study will assess the impact that reducing the number of doses of MenC vaccine given in the first few months of life has on the height and duration of the antibody response and on the B-cell memory response to the Hib-MenC vaccine given at 12 months of age. Given the possibility that further immunisations will become available for use in the UK infant immunisation schedule (e.g. against serogroup B meningocococcus^13^), a reduction in the number of MenC vaccines given in the first few months of life in the UK needs to be considered. The option of a single priming dose of MenC vaccine given in the first few months of life, followed by a booster dose of Hib-MenC at 12 months of age, may potentially enable both a reduction in infant MenC doses and the sustained protection afforded by ‘prime-boost’ immunisation schedules. It is therefore appropriate to compare this possibility with the current UK schedule (2 doses priming) and the option used in many other countries (single dose of MenC at 12 months of age with no priming).

There are, however, no published studies of a MenC immunisation programme using single infant priming followed by a booster dose of a MenC conjugate vaccine at 1 year of age. Several studies have assessed the immunogenicity of a single priming dose of MenC vaccine given at 2 months of age,^14,15^ or the antibody response after the first MenC dose,^16,17^ but none have assessed the impact of reducing the number of priming MenC vaccine doses on the response to the booster dose of Hib-MenC at 12 months of age. This is of particular relevance as it is likely that this booster dose response is of more importance in generating sustained population immunity against MenC disease than the response to the infant (priming) doses of MenC vaccine. Conversely, without the 12 month booster (the original UK schedule had no booster), antibody levels wane very rapidly and the majority of children have antibody levels below the protective threshold within a few years of immunisation. Indeed, recent studies have shown that the majority of UK primary school aged children (immunised with a single dose of MenC vaccine in the “catch up” campaign in 1999) have low antibody titres^18^ (figure 1).

**Figure 1**: Rates of seroprotection (SBA titres ≥ 1:8) against MenC disease in UK children and adolescents (adapted from Perrett et al (unpublished data) and Snape et al, 2008^18^ , showing that the vast majority of children under 10 years of age in the UK in 2008 are not protected against serogroup C meningococcal disease – these children received MenC either as a 3 dose infant schedule with no booster (children aged 5-10 years), or as a single dose of vaccine in the catch up campaign in 1999 (those now over 10 years of age).

Since MenC causes disease throughout childhood and adolescence^19^, and the effectiveness of MenC vaccines are known to decline in populations whose SBA titres have waned post MenC immunisation^20^, maintaining adequate antibody levels seems vital for individual immunity. Despite the central importance of this concept, there are no published systematically collected data on the persistence of immunity following immunisation in the current UK schedule.

Since MenC antibody titres are low among UK children under 10 years of age, the low levels of MenC disease currently seen in the UK may be attributed to herd immunity, which is most likely due to reduced MenC nasopharyngeal carriage rates in adolescents and young adults^21^. This herd immune effect was induced by the catch up campaign when the vaccine was introduced in 1999 and is related to the high effectiveness and antibody persistence in the older vaccinated groups (over 10 years) that effectively blocks transmission of the organism in the population, presumably because teenagers and young adults drive transmission of this organism. However, whilst herd immunity means that in 2009 there is very little risk of MenC disease even amongst the unimmunised, it seems unlikely that herd immunity will be maintained indefinitely, especially now that most children have very low antibody levels. In the UK the future maintenance of herd immunity is critically dependent on the persistence of the immune response following the 12 month booster dose of Hib-MenC, since no further doses of the MenC vaccine are currently given in the schedule.

The impact of reducing the number of priming MenC vaccine doses on the response to the 12 month booster dose of Hib-MenC therefore needs to be specifically assessed in an appropriately designed clinical trial as proposed herein.

Intriguingly, Borrow et al. have suggested that reducing the number of MenC priming doses from 2 to 1 may actually enhance the response to the 12 month booster dose^14^. In this study children received a dose of a 'plain polysaccharide' meningococcal serogroup A and C vaccine, rather than a conjugated MenC vaccine, at 12 months of age^14^. Children immunised with a single priming dose of MenC vaccine mounted a greater response to the 12 month dose of plain polysaccharide vaccine than those immunised with 2 or 3 infant doses of MenC vaccine, such that the single dose priming MenC group had the highest SBA geometric mean titres at 13 months of age (figure 2). If similar results were observed in response to the Hib-MenC conjugate vaccine, this would suggest that reducing the number of priming MenC vaccine doses may actually enhance the maintenance of immune protection through late childhood.

The investigators are not aware of any studies addressing the comparison between the number of priming doses and the response to the booster dose at 12 months of age. One currently recruiting study being conducted by the Health Protection Agency (HPA) is examining the important question of 1 dose priming schedules with MenC vaccines from different manufacturers, but the design of this study does not include a comparison of different priming schedules, and will not provide information on long term persistence of antibodies^22^.

**Figure 2**: MenC specific SBA geometric mean titres 1 month after immunisation with a MenAC plain polysaccharide vaccine at 12 months of age*, according to number of priming doses received in infancy (adapted from Borrrow et al^14^).

Single dose priming

Two dose priming

Three dose priming

* MenAC plain polysaccharide vaccine used in this study as an immunological challenge to assess immune priming. The vaccine currently used as a booster dose in the UK schedule is a combined Hib-MenC conjugate vaccine, for which no studies have assessed the impact of altering the number of priming doses of MenC vaccine. (Note that polysaccharide vaccines are not used in routine infant immunisation but no similar data are available for boosting with a MenC conjugate vaccine).

In addition, several studies have indicated that the response to the Hib-MenC-TT booster may depend on the type of MenC conjugate vaccine that is used for priming in infancy^23^. These differences may be related to the type of carrier protein that is used for the priming doses, or may be an effect of using different carrier proteins for priming and boosting. These previous studies have assessed the differences in schedules that had 2 or 3 priming doses; whereas this current study will be designed to assess potential differences in a single dose priming schedule. The two MenC conjugate vaccines that will be addressed are the two which are most commonly used in the UK and Europe: MenC-CRM_197_ (conjugated to mutant diphtheria toxin) and MenC-TT (conjugated to tetanus toxoid).

Furthermore, this proposed study will evaluate a novel means of assessing the induction of immunological memory, a defining feature of a successful conjugate vaccine^24^. Immune memory has classically been assessed by the anamnestic response to a plain polysaccharide boost. There are, however, uncertainties regarding the appropriateness of administering meningococcal plain polysaccharide vaccines to children <2 years of age who are known to respond poorly to unconjugated polysaccharide antigens^25^. In addition the hyporesponsiveness observed following repeated doses of plain polysaccharide vaccines raises concerns that receipt of these vaccines could potentially hinder a child’s ability to respond to natural infection with MenC. It has therefore recently been proposed that the WHO guidelines on the clinical evaluation of MenC vaccines be altered to recommend the use of booster doses of conjugate vaccines to assess immunologic memory^26^. However, no studies have provided data to allow distinction between primed and unprimed responses. One suggested means of doing this is to assess whether SBA titres increase above baseline more rapidly following a ‘challenge’ dose of MenC vaccine in those who have previously been primed by prior immunisation with a MenC vaccine than in those that are vaccine naïve. No clinical trials have previously assessed this, and the design of this study affords an opportunity to generate novel data to explore this issue. If it can be shown that a rise above baseline antibody levels can be seen at day 6 in primed, but not in unprimed, participants, this will provide an important new measure of immune priming that will be relevant for the design of future studies of conjugate vaccines.

*Alternating limb component*

This study would also provide the opportunity to investigate whether the immunogenicity of the Hib, tetanus and pneumococcal components, within the routinely recommended vaccines (Pediacel and Prevenar-13), that are administered concurrently with the MenC vaccine would be affected if sequential doses are administered within the same limb or in the alternating limb. Protection against vaccine preventable diseases is dependent on the generation of sustained and functional disease-specific antibody concentrations or a timely ‘secondary’ antibody response on exposure to the relevant disease which in turn are dependent on the generation of mature B cells in lymph nodes draining the site of vaccination. After priming, a significant proportion of mature B cells remain within these nodes^9^, accompanied by retained antigen and memory CD4 T-cells that can aid antibody responses^10^. Memory B cells appear to preferentially home to lymph nodes that have been primed with their cognate antigen^27^. Sequential immunisations, as performed in clinical trials, may therefore be more effective if given into the same limb since vaccine antigens should reach more memory B and T-cells, resident in draining lymph nodes. Post licensure sequential doses of vaccines are not usually administered in the same limb, a practice that was shown to result in reduced response rates to an intradermal rabies vaccine^28^. No further studies have been performed to assess the effect that alternating limbs with sequential immunisations has on the immunogenicity of conjugate vaccines.

***Immuno-genetics***

An additional aspect to be assessed in this study is the impact of genetic factors influencing the response to immunisation. Host immuno-genetics are likely to play a critical role in modulating the responses to paediatric vaccines. Twin studies on several vaccines including measles, mumps and rubella, have shown high heritability of vaccine antibody responses^29^. Some genetic associations have already been identified between genes of the adaptive and innate immune response and some vaccines, for example human leukocyte antigen (HLA) alleles and measles antibody responses^30,31^, and IL-1β polymorphisms and hepatitis B vaccine responses^32^. These studies have been small scale and based on single candidate genes and the extent to which genetic variation contributes to vaccine responses remains poorly understood. Insight into which genetic variants affect responses to specific vaccines will be of value for 2 main reasons:

1. it will help identify the critical immune pathways leading to protection after vaccination and lead to the production of more effective vaccines
2. it will help identify genes that may play important roles in wild-type infection and lead to better understanding of disease pathogenesis, which in turn may lead to the development of novel therapies

The blood samples obtained in this study provide an opportunity to extract DNA from the cellular plug remaining after serum centrifugation. The DNA samples obtained in this study can then contribute to a DNA bank pooling samples from multiple different Oxford Vaccine Group studies. These DNA samples can then be used for genome wide analysis of the genetic factors influencing the host response to the vaccines received in the relevant studies. This DNA extraction and storage will only occur with the specific consent of participants, and DNA will not be analysed for any other purpose than to assess factors influencing the immune response to vaccines.

# OBJECTIVES

The overall objective of this study is to determine the relative immunogenicity of different schedules of serogroup C meningococcal (MenC) vaccine in the first year of life and to determine whether a reduced dose schedule could be used to save costs for the NHS.

## 5.1 Primary Objective

## The primary objective of this study is to demonstrate non-inferiority of the geometric mean titres (GMTs) of meningococcal serogroup C (MenC) specific serum bactericidal antibodies, using rabbit complement (rSBA), 1 month after a 12 month dose of Hib-MenC vaccine in children receiving a single dose of MenC-CRM_197_vaccine at 3 months of age (Single Dose priming) compared with those receiving 2 doses at 3 and 4 months of age (Two Dose priming). Non-inferiority of the MenC serum bactericidal antibody geometric mean titres (SBA GMTs) would imply that the reduced schedule of MenC immunisation would be a more cost effective method of providing sustained immunity against MenC disease through childhood.

## 5.2 Secondary Objectives

Reduced dose MenC component:

1. To assess whether MenC SBA GMTs measured 1 month after a 12 month dose of Hib-MenC are higher in children previously receiving Single Dose MenC-CRM_197_ vaccine priming at 3 months of age (Single Dose MenC-CRM_197_ Group) than in those receiving no priming doses of MenC vaccine (Control Group), demonstrating whether single dose MenC priming offers any advantage over no priming in terms of antibody levels at 13 months, and whether a single infant dose of MenC vaccine induces immune memory.

2. In an exploratory analysis, to compare the MenC SBA GMTs at day 6 after the 12 month dose of vaccine in a subset of participants (64 participants each from the Single Dose MenC-CRM CRM_197_, Single Dose MenC-TT and Two Dose MenC Groups, and all 64 in the control group: n=256) from all four groups, as it has been proposed that assessment of specific antibody levels at this earlier time point will more effectively discriminate between 'primed' and 'unprimed' immune responses to vaccines.

3. To assess the MenC SBA GMTs 2 months after a dose of MenC-CRM_197_ vaccine at 3 months of age (Single Dose MenC-CRM CRM_197_ Group) compared to the MenC SBA GMTs taken 1 month after a course of MenC-CRM_197_ vaccine at 3 and 4 months of age (Two Dose MenC Group), and to a Control Group receiving no infant MenC immunisation who would be sampled at 5 months of age.

4. To assess MenC SBA GMTs 2 months after a dose of a MenC-TT vaccine at 3 months of age (Single Dose MenC-TT Group) compared to a Single Dose of a MenC-CRM_197_ conjugate vaccine (Single Dose MenC-CRM_197_ Group), and to the Control Group, on a blood sample taken at 5 months of age.

5. To assess MenC SBA GMTs measured 12 months after a 12 month dose of Hib-MenC in all groups receiving priming vaccines with MenC-CRM_197_, or no priming, to determine whether 2 dose MenC priming offers any advantage over single dose MenC priming or no priming in terms of antibody levels at 24 months.

6. To assess MenC SBA GMTs 12 months after a 12 month dose of Hib-MenC in children previously receiving a single dose of a MenC-TT vaccine at 3 months of age (Single Dose MenC-TT Group) compared to a single dose of a MenC-CRM_197_ conjugate vaccine (Single Dose MenC-CRM_197_ Group).

7. It is intended to measure the numbers of MenC specific memory B cells in the blood at 5 months, 12 months, 6 days following the 12 month booster dose, at 13 months and 24 months on a subset of participants. Specific memory B cells against diphtheria and tetanus will also be measured and used as a control. As many participants as possible would be included in this subset but the number would be determined by the practicalities of getting the blood to the laboratory in time (before midday) for processing.

8. To assess the local and systemic adverse reactions experienced by participants in the four different study groups after immunisation with each dose of the MenC-CRM_197_, MenC-TT and Hib-MenC vaccines.

Alternating limb component

1. To compare the *S. pneumonia*e IgG GMCs and percentage of infants with serum concentration of *S. pneumonia*e specific IgG ≥0.35 μg/ml for all 13 PCV13 serotypes at 5, 12, 13 and 24 months of age in children receiving PCV13 in a consistent limb (subgroups ‘a’) vs those receiving this in alternating limbs (subgroups ‘b’).
2. To compare serotype specific pneumococcal B cell phenotype in a subset of participants at 5, 12, 13 and 24 months of age in children receiving PCV13 in a consistent limb (sub groups ‘a’) vs those receiving this in alternating limbs (subgroups ‘b’).

3. To compare the anti-PRP IgG GMCs and percentage of infants with serum concentration of anti-PRP IgG ≥0.15μg/ml and ≥1.0 μg/ml and at 5, 12, 13 and 24 months of age in children receiving DTaP-IPV-Hib in a consistent limb (sub groups ‘a’) vs those receiving this in alternating limbs (subgroups ‘b’).

4. To compare the anti-tetanus toxoid GMCs and percentage of infants with anti-tetanus toxoid >0.1 IU/ml at 5,12, 13 and 24 months of age in children receiving DTaP-IPV-Hib in a consistent limb (sub groups ‘a’) vs those receiving this in alternating limbs (subgroups ‘b’)

5. To compare the local or systemic vaccine reactions in subgroups a and b at each vaccination time point.

**6. TRIAL DESIGN**

6.1 Summary of Trial Design

This is a phase IV open label randomised controlled trial enrolling 498 participants. Study participation would be for 23-24 months during which there will be 8 study visits as outlined in the attached figure and as detailed in section 6.4.2. A list of the study procedures and the interval between study visits are shown in Appendices A, B and C. Study visit 6 would be performed in all participants in the control group, and an equal number (i.e. 64) of participants in the Two Dose MenC, Single Dose MenC-CRM_197_ and Single Dose MenC-TT groups (as determined by randomisation at enrolment).

***Two dose MenC Group** **n=160**

MenC-CRM_197_ at 3 & 4 months

Visit 4

Visit 1

***Single dose MenC-CRM_197_ Group n=160**

MenC-CRM_197_ at 3 months

Visit 5

Visit 6**

Visit 7

Hib-MenC boost + PCV13 at 12 months

VP

VP

VP

VP

*Control group n=64

Visit 2

Visit 3

MMR

Visit 8

VP

*Single dose MenC-TT Group n=114 MenC-TT at 3 months

*Participants would be randomised to receive MenC-CRM_197_ at 3 and 4 months (Two Dose MenC Group), or a single dose at 3 months (Single Dose MenC-CRM_197_ Group) or a single dose of MenC-TT at 3 months (Single dose MenC-TT Group) or no priming doses (Control Group). All participants will receive the DTaP-IPV-Hib vaccine at 2, 3 and 4 months and the PCV13 at 2 and 4 months according to the UK immunisation schedule. All participants would be boosted with the Hib-MenC vaccine and the booster dose of PCV13 at 12 months and will receive the MMR vaccine at 13 months.

Venepuncture (VP), taking 5 ml of blood, will be performed at 5 months (Visit 4), and taking 7.5ml of blood will be performed at 12 months (Visit 5), 12 months+6 days (Visit 6), at 13 months (Visit 7) and at 24 months (Visit 8). Two attempts may be made to obtain a blood sample at each visit, if the parent gives verbal consent at the time of the procedure for a second attempt. If no blood is obtained, a second visit may be made if the parent agrees to this.

** Visit 6 will be performed on all participants in the control group and a subset of 64 participants in the Two Dose MenC, Single Dose MenC-CRM_197_ and Single Dose MenC-TT groups (as determined by randomisation at enrolment, see section 6.4.4)

In addition, participants will be randomised at enrolment to receive PCV13 and DTaP-IPV-Hib in either consistent limbs (subgroup a) or alternating limbs (subgroup b) as follows

| **Subgroup a**  (Consistent limbs) | **Subgroup b**  (Alternating limbs) |
| --- | --- |
| DTaP-IPV-Hib in right leg at 2, 3 and 4 months  PCV13 in right leg at 2, 4and 12 months | DTaP-IPV-Hib in left leg at 2 months and in right leg at 3 and 4 months*  PCV13 in left leg at 2 months, right leg at 4 months and left arm at 12 months |

## * DTaP-IPV-Hib given in same limb at 3 and 4 months to maintain consistency of co-administration of vaccines in the same limb (i.e. MenC-CRM_197_ or MenC-TT always given by itself and DTaP-IPV-Hib co-administered with PCV13 at 2 and 4 months – see Appendix A).

## 6.2 Primary and Secondary Endpoints/Outcome Measures

Primary endpoint

The difference in the MenC rSBA GMTs between the participants primed with two doses of MenC-CRM_197_ (Two Dose MenC Group) and with one dose of MenC-CRM_197_ (Single Dose MenC-CRM_197_ Group), one month following the Hib-MenC booster dose at 12 months.

Secondary endpoints

*Reduced MenC component*

After administration of the Hib-MenC booster dose at 12 months of age the following comparisons would be performed in order to assess differences between the:

1) MenC rSBA GMTs between the Two Dose MenC Group and the Control group at 6 and 28 days and 12 months later

2) MenC rSBA GMTs between the Single dose MenC-CRM_197_ group and the Control group at 6 and 28 days and 12 months later

3) MenC rSBA GMTs between the Single Dose MenC-TT group and the Control group at 6 and 28 days and 12 months later

4) MenC rSBA GMTs between the Single Dose MenC-CRM_197_ group and the Single Dose MenC-TT group at 6 and 28 days and 12 months later

5) Percentage of participants with MenC rSBA ≥1:8 and ≥1:128 between the Two Dose MenC group and the Control group at 6 and 28 days and 12 months later

6) Percentage of participants with MenC rSBA ≥1:8 and ≥1:128 between the Single dose MenC-CRM_197_ group and the Control group, at 6 and 28 days and 12 months later

7) Percentage of participants with MenC rSBA ≥1:8 and ≥1:128 between the Single dose MenC-TT group and the Control group, at 6 and 28 days and 12 months later

8) Percentage of participants with MenC rSBA ≥1:8 and ≥1:128 between the Single Dose MenC-TT group and the Control group at 6 and 28 days and 12 months later (Group 4 vs Group 3)

9) Percentage of participants with MenC rSBA ≥1:8 and ≥1:128 between the Single Dose MenC-CRM_197_ group and the Single Dose MenC-TT group at 6 and 28 days and 12 months later

10) Percentage of participants with MenC rSBA GMTs >1000 between the Two Dose MenC Group and the Control Group, at 6 and 28 days and 12 months later.

11) Percentage of participants with MenC rSBA GMTs >1000 between the Single Dose MenC-CRM_197_ Group and the Control Group, at 6 and 28 days and 12 months later.

12) Percentage of participants with MenC rSBA GMTs >1000 between the Two Dose MenC Group and the Single Dose MenC-CRM_197_ Group, at 6 and 28 days and 12 months later.

13) Percentage of participants with MenC rSBA GMTs >1000 between the Single Dose MenC-TT group and the Control group at 6 and 28 days and 12 months later (Group 4 vs Group 3)

14) Percentage of participants with MenC rSBA GMTs >1000 between the Single Dose MenC-TT Group and the Control Group, at 6 and 28 days and 12 months later.

After administration of the last MenC-CRM_197_ dose at 4 months of age the following comparisons would be performed to assess differences between:

15) MenC rSBA GMTs between the Two Dose MenC Group vs Single Dose MenC-CRM_197_ Group, Two Dose MenC Group vs Control Group, Single Dose MenC-CRM_197_ vs Control Group, Single Dose MenC-TT vs Control group, and Single Dose MenC-CRM_197_ Group vs Single Dose MenC-TT group at 5 and 12 months of age.

16) Percentage of participants with MenC rSBA ≥1:8 between the Two Dose MenC Group vs Single Dose MenC-CRM_197_ Group, Two Dose MenC Group vs Control Group, Single Dose MenC-CRM_197_ vs Control Group, Single Dose MenC-TT vs Control Group, Single Dose MenC-CRM_197_ Group vs Single Dose MenC-TT Group at 5 and 12 months of age.

17) Percentage of participants with MenC rSBA ≥1:128 between the Two Dose MenC Group vs Single Dose MenC-CRM_197_ Group, Two Dose MenC Group vs Control Group, Single Dose MenC-CRM_197_ vs Control Group, Single Dose MenC-TT vs Control Group, Single Dose MenC-CRM_197_ Group vs Single Dose MenC-TT group at 5 and 12 months of age.

18) Number of MenC memory B cells at 5 months, 12 months, 12 months+6 days, 13 months and 24 months (on a subset of participants) between the Two Dose MenC Group vs Single Dose MenC-CRM_197_ Group, Two Dose MenC Group vs Control Group, Single Dose MenC-CRM_197_ vs Control Group, Single Dose MenC-TT vs Control Group, Single Dose MenC-CRM_197_ Group vs Single Dose MenC-TT.

Alternating limb component

After administration of the DTaP-IPV-Hib and PCV-13 the following comparisons would be performed to assess differences between the:

1. Anti-*S. pneumoniae* IgG GMCs or percentage of infants with serum concentration of *S. pneumonia*e specific IgG ≥0.35 μg/ml for each of the 13 serotypes at 5, 12, 13 and 24 months of age in children receiving PCV13 in a consistent limb (sub groups ‘a’) vs those receiving this in alternating limbs (subgroups ‘b’).
2. Serotype specific pneumococcal B cell phenotype in a subset of participants at 5, 12, 13 and 24 months of age in children receiving PCV13 in a consistent limb (sub groups ‘a’) vs those receiving this in alternating limbs (subgroups ‘b’).

3. Anti-PRP IgG GMCs or percentage of infants with serum concentration of anti-PRP IgG ≥ 0.15 μg/mL and ≥1.0 μg/ml at 5, 12, 13 and 24 months of age in children receiving DTaP-IPV-Hib in a consistent limb (sub groups ‘a’) vs those receiving this in alternating limbs (subgroups ‘b’).

4. Anti-tetanus toxoid GMCs or percentage of participants with anti-tetanus toxoid >0.1IU/ml at 5, 12, 13 and 24 months of age in participants in receiving DTaP-IPV-Hib in a consistent limb (sub groups ‘a’) vs those receiving this in alternating limbs (subgroups ‘b’).

*Reactogenicity*

The following comparisons would be performed to assess differences between:

Local adverse events

1. The percentage of infants with each type of adverse event, and at least one adverse event for local adverse events after one dose of MenC-CRM_197_ at 3 months (Single Dose MenC-CRM_197_ Group) vs the second dose of MenC-CRM_197_ at 4 months (Two Dose MenC Group).
2. The percentage of infants with each type of adverse event, and at least one adverse event for local adverse events after one dose of MenC-CRM_197_ at 3 months (Single Dose MenC-CRM_197_ Group) vs one dose of MenC-TT at 3 months (Single Dose MenC-TT Group).
3. The percentage of infants with each type of adverse event, and at least one adverse event for local adverse events after each dose of DTaP-IPV-Hib (at 2, 3 and 4 months) and PCV13 (at 2 and 4 months) in the consistent limb group (sub groups ‘a’) vs. the alternating limb group (subgroups ‘b’).
4. The percentage of infants with each type of adverse event, and at least one adverse event for local adverse events after the 12 month booster MenC and PCV13 vaccination in the
   1. Two Dose MenC Group vs 0 Dose Control Group
   2. Single Dose MenC-CRM_197_ Group vs. 0 Dose Control Group
   3. Single Dose MenC-TT Group vs. 0 Dose Control Group
   4. Single Dose MenC-CRM_197_ Group vs. Two Dose MenC Group
   5. Single Dose MenC-CRM_197_ Group vs Single Dose MenC-TT Group
   6. the consistent limb group vs. the alternating limb group

Systemic adverse events

1. The percentage of infants with each type of adverse event, and at least one adverse event for systemic adverse events after MenC (3 and 4 months), DTaP-IPV-Hib (2,3 and 4 months) and PCV13 (2 and 4 months) vaccination in the:
   1. Two Dose MenC Group vs. 0 Dose Control Group
   2. Single Dose MenC-CRM_197_ Group vs. 0 Dose Control Group
   3. Single Dose MenC-TT Group vs. 0 Dose Control Group
   4. Single Dose MenC-CRM_197_ Group vs. Two Dose MenC Group
   5. Single Dose MenC-CRM_197_ Group vs Single Dose MenC-TT Group
   6. The consistent limb group vs. the alternating limb group
2. The percentage of infants with each type of adverse event, and at least one adverse event for systemic adverse events after the 12 month booster MenC and PCV13 vaccination in the:
   1. Two dose MenC Group vs. 0 dose control Group
   2. Single Dose MenC-CRM_197_ Group vs. 0 Dose Control Group
   3. Single Dose MenC-TT Group vs. 0 Dose Control Group
   4. Single Dose MenC-CRM_197_ Group vs. Two Dose MenC Group
   5. Single Dose MenC-CRM_197_ Group vs Single Dose MenC-TT Group
   6. The consistent limb group vs. the alternating limb group

6.3 Trial Participants

## 6.3.1 Overall Description of Trial Participants

Healthy 6-12 week old male and female infants born between 37 and 42 weeks of gestation would be recruited in four centres in the United Kingdom (Oxford, London, Bristol and Southampton) and one centre in Malta.

## 6.3.2 Inclusion Criteria

- Healthy male or female infants aged 6-12 weeks at the time of the first vaccination and who were born between 37 and 42 weeks of gestation
- Infants who are known to be free from medical problems as determined by a medical history and clinical examination
- Parents or guardians who are willing for their child to participate and who would be expected to comply with the requirements of the protocol
- Parents/guardians who have given informed consent for their child’s participation in the study

6.3.3 Exclusion Criteria

The participant may not enter the study if ANY of the following apply:

- History of invasive meningococcal C disease
- Previous vaccination against meningococcal serogroup C disease
- Planned administration/administration of vaccines, since birth, other than the study vaccines (with the exception of oral rotavirus vaccine, Hepatitis B vaccine, Hepatitis A vaccine, Influenza vaccines and BCG, that can be administered 14 days before or after study vaccines and Varicella vaccine that can be administered 14 days before or after study vaccines or 4 weeks before or after other live vaccines, i.e. MMR. Varicella vaccine can also be given in the form of the combined Measles Mumps Rubella Varicella vaccine from 13 months of age).
- Receipt of investigational vaccines/drugs, other than the vaccines used in the study, within 30 days prior to receiving the first dose of the vaccines or their planned use during the study period, until 1 month after the administration of the final study vaccine (ie at 12 months of age).
- Confirmed or suspected immunosuppressive or immunodeficient conditions, including human immunodeficiency virus (HIV) infection.
- A family history of congenital or hereditary immunodeficiency.
- Receipt of more than 2 weeks of immunosuppressants or immune modifying drugs, (e.g. prednisolone >0.5mg/kg/day)
- History of allergy to any component of the vaccines.
- Major congenital defects or serious chronic illness.
- History of any neurologic disorders or seizures
- Acute disease at the time of recruitment as defined by the presence of a moderate or severe illness with or without fever (with the exception of minor illnesses such as diarrhoea, mild upper respiratory infection without fever). In such situations enrolment should be postponed until the participant has recovered.
- Administration of immunoglobulins and/or any blood products since birth or planned administration during the study period
- Parents who plan to move out of the geographical area where the study would be conducted.

## 6.3.4 Elimination criteria during the study

The following criteria will be checked at each visit subsequent to the first visit and if any become applicable during the study, it will not require withdrawal of the participant from the study but may determine the participant’s evaluability in the completer’s population (CP) analysis, in which case the data would be included in the Intention to Treat (ITT) analysis.

- Use of any investigational or non-registered product (drug or vaccine) other than the study vaccine(s) during the study period, until 1 month after the administration of the final study vaccine (ie at 12 months of age).
- Chronic administration (defined as more than 14 days) of immunosuppressants or other immune-modifying drugs during the study period. (For corticosteroids, this will mean prednisone, or equivalent, ≥ 0.5 mg/kg/day. Inhaled and topical steroids are allowed.)
- Administration of a vaccine not foreseen by the study protocol during the period starting from 30 days before each dose of vaccine(s) and ending 30 days after.
- Administration of immunoglobulins and/or any blood products during the study period.
- Administration of any of the vaccines used in the study outside of the stipulated time period
- Administration of any of the vaccines used in the study in an incorrect limb

### 6.3.5 Delaying criteria during the study visits

Participants with an acute illness would be recruited after the illness has resolved.

Vaccine administration will be delayed in case of acute illness or axillary temperature >38^o^C.

Venesampling will be delayed for 1 week after the stopping of antibiotics in order to avoid interference with the MenC rSBA assays.

### 6.3.6 Study Procedures

The schedule of the following procedures has been listed in Appendix B.

**Detailed description of study visits**

**i) Visit 1: Study Day 0: First vaccination visit (6-12 weeks)**

- Written informed consent is obtained from the participant’s parent/guardian.
- Inclusion/exclusion criteria will be checked prior to enrolment.
- A medical and vaccination history will be taken and recorded
- Any concomitant medications which are not listed in the exclusion criteria and thus allowed by the study protocol will be recorded.
- Pre-vaccination assessment of body temperature
- Randomisation
- Vaccination: intramuscular administration of one dose of the vaccines used in the study (according to group) will be administered as described in section 7.
- Explanation of diary cards to assess local and systemic adverse events for 4 days following vaccination

The vaccinees will be observed closely for at least 15 minutes, with appropriate medical treatment readily available in case of a rare anaphylactic reaction following the administration of vaccines.

The subjects’ parents/guardians will be instructed to contact the investigator immediately should they manifest any signs or symptoms they perceive as serious.

**ii) Visit 2: Study Month 1: Vaccination visit, at approximately 3 months of age (28-42 days after visit 1)**

- Reporting of SAEs that might have occurred since the last visit.
- Collection of diary card
- Check of elimination criteria.
- Check of contraindications.
- Recording of any concomitant immunosuppressive medication or vaccines not foreseen by the study protocol
- Pre-vaccination assessment of axillary body temperature.
- Vaccination: intramuscular administration of one dose of the vaccines used in the study (according to group) will be administered.
- Explanation of diary card to assess local and systemic adverse events for 4 days following vaccination

The vaccinees will be observed closely for at least 15 minutes, with appropriate medical treatment readily available in case of a rare anaphylactic reaction following the administration of vaccines.

The subjects’ parents/guardians will be instructed to contact the investigator immediately should they manifest any signs or symptoms they perceive as serious.

**iii) Visit 3: Study Month 2: Vaccination visit, at approximately 4 months of age (28-42 days after Visit 2)**

- Reporting of SAEs that might have occurred since the last visit.
- Collection of diary card
- Check of elimination criteria.
- Check of contraindications.
- Recording of any concomitant immunosuppressive medication or vaccines not foreseen by the study protocol
- Pre-vaccination assessment of axillary body temperature
- Vaccination: intramuscular administration of one dose of the vaccines used in the study (according to group) will be administered.
- Explanation of diary card to assess local and systemic adverse events for 4 days following vaccination

The vaccinees will be observed closely for at least 15 minutes, with appropriate medical treatment readily available in case of a rare anaphylactic reaction following the administration of vaccines.

The subjects’ parents/guardians will be instructed to contact the investigator immediately should they manifest any signs or symptoms they perceive as serious.

**iv) Visit 4: Study Month 3: Blood sampling visit, at approximately 5 months of age (28-42 days after visit 3)**

- Reporting of SAEs that might have occurred since the last visit.
- Collection of diary card
- Check of elimination criteria.
- Recording of any concomitant immunosuppressive medication or vaccines not foreseen by the study protocol
- Collection of blood for serology (and B cells studies on a subset of participants): 5.0 ml of whole blood. If less than 4mL of blood is collected, the whole sample will be used serology assays. If 4-5mL of blood is collected the sample will be split in half and shared evenly between serology assays and B cell studies. 2-2.5mL whole blood will provide about 1mL of serum for serology.

The subjects’ parents/guardians will be instructed to contact the investigator immediately should they manifest any signs or symptoms they perceive as serious.

**v) Visit 5: Study Month 10: Vaccination visit, at 12 months of age (51 – 58 weeks)**

- Check of exclusion criteria.
- Check of elimination criteria.
- Check of contraindications.
- Assessment and recording of medical history and vaccination history.
- Recording of any concomitant medication or vaccines not foreseen by the study protocol
- Retrospectively reporting of SAEs that might have occurred since the last visit of the primary phase.
- Pre-vaccination assessment of body temperature
- Collection of blood for serology (and B cell studies on a subset of participants): 7.5 ml of whole blood. If less than 4mL of blood is collected, the whole sample will be used serology assays. If 4-6mL of blood is collected the sample will be split in half and shared evenly between serology assays and B cell studies. If >6mL of blood is collected, 3mL will be used to provide at least 1mL of serum for serology assays, and the remainder will be used for B cell studies.
- Vaccination: intramuscular administration of one dose of the Hib-MenC vaccine and one dose of PCV13
- Explanation of diary card to assess local and systemic adverse events for 4 days following vaccination

The vaccinees will be observed closely for at least 15 minutes, with appropriate medical treatment readily available in case of a rare anaphylactic reaction following the administration of vaccines.

The subjects’ parents/guardians will be instructed to contact the investigator immediately should they manifest any signs or symptoms they perceive as serious.

**vi)** **Visit 6: Study month 10: Blood sampling visit, at 6 days following Hib-MenC booster vaccination (Performed on 256 participants; all participants in the control group and 64 participants each from the Two Dose MenC, Single Dose MenC-CRM_197_ and Single Dose MenC-TT Groups as determined by randomisation at enrolment).**

- Reporting of SAEs that might have occurred since the last visit.
- Collection of diary card
- Collection of blood for serology (and B cell studies on a subset of participants): 7.5 ml of whole blood. If less than 4mL of blood is collected, the whole sample will be used serology assays. If 4-6mL of blood is collected the sample will be split in half and shared evenly between serology assays and B cell studies (if applicable). If >6mL of blood is collected, 3mL will be used to provide at least 1mL of serum for serology assays, and the remainder will be used for B cell studies (if applicable).

**vii) Visit 7: Study month 11: Blood sampling visit at approximately 13 to 14 months, 28-42 days after booster vaccination**

- Reporting of SAEs that might have occurred since the Visit 5.
- Recording of any concomitant immunosuppressive medication or vaccines not foreseen by the study protocol
- Collection of blood for serology (and B cell studies on a subset of participants): 7.5 ml of whole blood. If less than 4mL of blood is collected, the whole sample will be used serology assays. If 4-6mL of blood is collected the sample will be split in half and shared evenly between serology assays and B cell studies. If >6mL of blood is collected, 3mL will be used to provide at least 1mL of serum for serology assays, and the remainder will be used for B cell studies.
- Administration of MMR

The vaccinees will be observed closely for at least 15 minutes, with appropriate medical treatment readily available in case of a rare anaphylactic reaction following the administration of vaccines.

viii) **Visit 8:** **Study month 22: Blood sampling visit at approximately 23 to 24 months, 11-12 months after booster vaccination**

- Reporting of SAEs that might have occurred since the Visit 7.
- Recording of any concomitant immunosuppressive medication or vaccines not foreseen by the study protocol
- Collection of blood for serology (and B cell studies on a subset of participants): 7.5 ml of whole blood. If less than 4mL of blood is collected, the whole sample will be used serology assays. If 4-6mL of blood is collected the sample will be split in half and shared evenly between serology assays and B cell studies. If >6mL of blood is collected, 3mL will be used to provide at least 1mL of serum for serology assays, and the remainder will be used for B cell studies.
- Study conclusion

**6.4.1 Informed Consent**

The parents/legal guardians of the participants must personally sign and date the informed consent form before any study specific procedures are performed. Written and verbal versions of the participant information and Informed consent will be presented to the participants detailing the exact nature of the study; the implications and constraints of the protocol; the known side effects and any risks involved in taking part. It will be clearly stated that the parent/legal guardian is free to withdraw his/her child from the study at any time for any reason without prejudice to future care, and with no obligation to give the reason for withdrawal. Specific consent will be obtained for the genetic analysis to be performed in the study; consent for this may be refused and the participant will still be eligible to participate in the other aspects of the study.

### Parents/legal guardians will be allowed at least 24 hours to consider the information, and the opportunity to question the Investigator, their GP or other independent parties to decide whether they will participate in the study. Written Informed Consent will then be obtained by means of a parent/guardian dated signature and dated signature of the person who presented and obtained the informed consent. The person who obtained the consent would be suitably qualified and trained doctor or research nurse, and has been authorised to do so by the Chief/Principal Investigator. A copy of the signed Informed Consent will be given to the participants. The original signed form will be retained at the study site.

### 6.4.2 Screening and Eligibility Assessment

This is a multi- centre study which is planned to be conducted by the Oxford Vaccine Group, Bristol Children’s Vaccine Centre, Southampton University Hospital, and St George’s Vaccine Institute in the UK and the Malta Children’s Vaccine Group in Malta. An invitation letter, which describes the study and which includes a reply form, would be sent to parents. Parents who are happy to take part are requested to contact the local study team and would then be given the opportunity to discuss the study. An appointment would then be set up for parents willing for their child to take part in the study.

In the UK, eligible participants will be identified through the child health computers of the Primary Care Trusts. Investigators or other allied health professionals at the research sites may also approach parents/carers opportunistically on post-natal wards. Investigators may also ask GPs and Health Visitors within their local network to identify and approach potential parents/carers, who have children of suitable age to participate in the study during the enrolment period. Children of parents interested in participating in the study would then be visited at their homes and recruited to the study after obtaining informed consent.

In Malta eligible participant will be identified through the Birth Register held at Mater Dei Hospital. Children of parents interested in taking part in the study would be given an appointment at the Paediatric Day Care Unit in Mater Dei Hospital and recruited after obtaining informed consent.

Inclusion and exclusion criteria (specified in section 6.3.2 and 6.3.3) will be checked on the first visit during which enrolment and randomisation will take place. Screening procedures are detailed in section 6.4.

The following data will be documented on the CRFs:

Demographics

The date of birth, gender, and ethnicity will be recorded.

Medical History

Details of any history of disease or surgical interventions in all systems will be recorded:

Physical Examination

Axillary temperature will be recorded.

### 6.4.3 Baseline Assessments

Measurement of axillary temperature together with a check of the inclusion and exclusion criteria to determine if the infant is healthy will be performed.

### 6.4.4 Randomisation and Code-breaking

Randomisation for all UK and Maltese participants will take place at the Oxford Vaccine Group in the UK on a 10:10:7:4 basis to the four study groups (Two Dose MenC, Single Dose MenC-CRM_197_, Single Dose MenC-TT and Control Group). Each study group would then be randomised on a 1:1 basis to the consistent or alternate limb subgroup (a and b). 64 children from each of the Two Dose MenC, Single Dose MenC-CRM_197_ and Single Dose MenC-TT groups will also be randomly selected, as well as all the children in the Control group, to have the 12 month + 6 day blood visit, . Randomisation will occur by means of opening in a sequential manner a sealed envelope containing the study group the child is to be randomised to. These envelopes will be prepared by the study statistician and the list held by the statistician and the study sites.

Due to the nature of the study, the study is unblinded, therefore no unblinding procedure is required. Analysis of immunogenicity and safety data will be performed by study staff and laboratory staff in linked-anonymised form such that lab samples and diary cards will only be labelled with participant initials/number. In the event of twins being enrolled from the same family, the diary card may be labelled with the participant’s first initial or name.

### 6.4.5 Subsequent assessments

A detailed description of subsequent assessments may be found in section 6.3.6.

## 6.5 Definition of End of Trial

The end of trial is the date of completion of all study assays and after administration of any booster doses of vaccines that may be necessary for participants who have sub-optimal results.

## 6.6 Discontinuation/ Withdrawal of Participants from Study Treatment

Each participant has the right to withdraw from the study at any time. In addition, the investigator may discontinue a participant from the study at any time if the investigator considers it necessary for any reason including:

Ineligibility (either arising during the study or retrospective having been overlooked at screening)

Significant protocol deviation

Significant non-compliance with study requirements

An adverse event which requires discontinuation of the study medication or results in inability to continue to comply with study procedures

Consent withdrawal

Lost to follow up

If a participant is withdrawn from the study the blood samples and any safety data already collected would be included in the analysis.

The reason for withdrawal will be recorded in the CRF.

If the participant is withdrawn due to an adverse event, the investigator will arrange for follow-up visits or telephone calls until the adverse event has resolved or stabilised.

## 6.7 Source Data

In this study the CRF will be used as the source document for collection of demographic data, documentation of inclusion and exclusion criteria, medical and vaccination history and the findings on physical examination. If a participant sustains a SAE then hospital records would be accessed after obtaining parental/guardian consent. Identifying information would be stored separately from the CRF.

All documents will be stored safely in locked cupboards during the study and archived for 3 years after the child turns 18 years of age. Access to these documents would only be available to the investigators, monitors and auditors directly involved in the study. On all study-specific documents, other than the signed consent, the participant will be referred to by the study participant number/initials, not by name.

# TREATMENT OF TRIAL PARTICIPANTS

## 7.1 Description of Study Treatment

This is a Phase IV clinical trial and all vaccines used in this study are licensed.

1. **MenC-CRM_197_: (*Menjugate*, Novartis Vaccines and Diagnositics):** conjugate *Neisseria meningitidis* serogorup C polysaccharide-protein conjugate vaccine^33^.
2. **MenC-TT: (*NeisVac-C*, Baxter Vaccines):** conjugate *Neisseria meningitidis* serogorup C polysaccharide-protein conjugate vaccine^34^.
3. **DTaP-IPV-Hib: (*Pediacel*, Sanofi Pasteur, MSD):** combined diphtheria, tetanus, acellular pertussis, inactivated polio, *Haemophilus influenzae* type b (DTPa-IPV-Hib) vaccine^35^.
4. **PCV13: (*Prevenar-13*, Wyeth Vaccines):** thirteen valent pneumococcal polysaccharide-protein conjugate vaccine^36^.
5. **Hib-MenC: (*Menitorix*, GlaxoSmithKline Biologicals):** combined *Haemophilus influenzae* type b and *Neisseria meningitidis* serogroup C polysaccharide-protein conjugate vaccine^37^.

A measles mumps and rubella (MMR) vaccine will be administered during visit 7 but will not form part of the study evaluation.

## 7.1.1 Dosage and administration

Details on the dosage and administration of each vaccine are given in the study flow chart in Appendix A.

The vaccinees will be observed closely for 15 minutes following the administration of vaccines, with appropriate medical treatment readily available in case of a rare anaphylactic reaction.

Vaccination will be performed by a registered doctor or nurse.

**Injection technique**

In order to ensure proper intramuscular injection of the study vaccines, a 23G (0.5mm in diameter) needle of at least 1 inch (2.54 cm) length will be used. All vaccines will be administered intramuscularly. The following injection technique will be used^38^.

**Injections in the thigh**

The needle is inserted in the anterolateral aspect of the middle third of the thigh in the vastus lateralis muscle at an angle of 90^o^ to the long axis of the leg with the subject supine or whilst held in the ‘cuddle’. If more than 1 injection is to be given in to the thigh then they will be given at least 2.5 cm apart.

**Injections in the deltoid**

The injection in the deltoid will be done using a 25 mm (1 inch), 23 gauge needle. The needle is inserted in the deltoid at 90^o^ to the long axis of the arm.

- 1. **Storage of Study Treatment**

UK

Vaccines are kept in a locked room in each of the study centres. The refrigerator temperature is regularly monitored and any temperature deviation outside the +2^o^C to +8^o^C will be communicated to a designated person via a pager system. In the case of refrigerator failure all vaccines will be transferred to a backup vaccine fridge.

Malta

Vaccines are kept locked in a fridge in a locked room at the Paediatric Day Care Unit in Mater Dei Hospital. The refrigerator temperature is regularly monitored and any temperature deviation outside the +2^o^C to +8^o^C will be communicated to the chief engineer in hospital via the Building Management System. The chief engineer would then inform a designated study member via a pager system. In the case of refrigerator failure all vaccines will be transferred to a backup vaccine fridge.

## 7.3 Compliance with Study Treatment

Participants are vaccinated during the designated vaccine visits.

## 7.4 Accountability of the Study Treatment

*Menjugate, NeisVac-C, Pediacel, Prevenar-13, Menitorix* and the MMR vaccine will be supplied by Movianto to the each of the UK study sites. The Oxford Vaccine Group would supply all vaccines to the Malta site. All unused vaccines and returned used vials will be retrieved at the end of the study. The vaccines will be collected from the vaccine fridge at the beginning of each day scheduled for study visits. All used vaccine boxes will be returned to OVG at the end of each study day.

## 7.5 Concomitant Medication

Throughout the study the investigators will not prescribe any concomitant medications (with the possible exception of local anaesthetic cream if required for venepuncture). Only healthy infants will be recruited in the study and if the participant receives any of the vaccines/medications included in the exclusion criteria then the participant will not be enrolled but if already enrolled would be included in the ITT analysis but excluded from the according to protocol (ATP) analysis.

The following medications are contraindicated:

1) Concomitant vaccinations, except the study vaccines, rotavirus vaccine, the Hepatitis A and B vaccines, Influenza vaccines, BCG and the Varicella vaccine, that can be administered 14 days before or after study vaccines. (The Varicella vaccine can also be administered 4 weeks before or after other live vaccines, i.e. MMR. Varicella vaccine can also be given in the form of the combined Measles Mumps Rubella Varicella vaccine from 13 months of age).

2) Immunosuppressants e.g. azathioprine, cyclosporine, or prednisolone at a dose of >0.5mg/kg./day or >20mg/day for more than 2 weeks

3) Immunoglobulins and/or any blood product

**8. SAFETY REPORTING**

## 8.1 Definitions

### 8.1.1 Adverse Event (AE)

An AE or adverse experience is:

Any untoward medical occurrence in a patient or clinical investigation participant administered a medicinal product, which does not necessarily have to have a causal relationship with this treatment (the study medication). Since the medicinal products used in this study are vaccines the definition of the specific adverse events of fever and local reactions will follow the Brighton collaboration guidelines^39-42^.

An AE can be any unfavourable and unintended sign (including an abnormal laboratory finding), symptom or disease temporally associated with the use of the study medication, whether or not considered related to the study medication.

### 8.1.2 Adverse Reaction (AR)

All untoward and unintended responses to a medicinal product related to any dose.

The phrase "responses to a medicinal product" means that a causal relationship between a study medication and an AE is at least a reasonable possibility, i.e., the relationship cannot be ruled out.

All cases judged by either the reporting medically qualified professional or the sponsor as having a reasonable suspected causal relationship to the study medication qualify as adverse reactions.

### 8.1.3 Severe Adverse Events

To ensure no confusion or misunderstanding of the difference between the terms "serious" and "severe", which are not synonymous, the following note of clarification is provided:

The term "severe" is often used to describe the intensity (severity) of a specific event (as in mild, moderate, or severe myocardial infarction); the event itself, however, may be of relatively minor medical significance (such as severe headache). This is not the same as "serious," which is based on patient/event outcome or action criteria usually associated with events that pose a threat to a participant's life or functioning. Seriousness (not severity) serves as a guide for defining regulatory reporting obligations.

### 8.1.4 Serious Adverse Event or Serious Adverse Reaction

A serious adverse event or reaction is any untoward medical occurrence that at any dose:

- Results in death,
- Is life-threatening,

NOTE: The term "life-threatening" in the definition of "serious" refers to an event in which the participant was at risk of death at the time of the event; it does not refer to an event which hypothetically might have caused death if it were more severe.

- Requires inpatient hospitalisation or prolongation of existing hospitalisation,
- Results in persistent or significant disability/incapacity, or
- Is a congenital anomaly/birth defect.
- Other important medical events*

*Other events that may not result in death, are not life threatening, or do not require hospitalisation, may be considered a serious adverse event when, based upon appropriate medical judgement, the event may jeopardise the patient and may require medical or surgical intervention to prevent one of the outcomes listed above.

### 8.1.5 Expected Serious Adverse Events/Reactions

No serious adverse events are expected from the vaccines administered in this study. All vaccines used are already licensed and used in several European countries.

### 8.1.6 Suspected Unexpected Serious Adverse Reactions

A serious adverse reaction, the nature or severity of which is not consistent with the summary of product characteristics.

### 8.1.7 Medically Significant adverse events

An adverse event that results in a consultation with a physician or other health professional

## 8.2 Reporting Procedures for All Adverse Events

All AEs attributed to the administration of DTaP-IPV-Hib, MenC-CRM_197_, MenC-TT, PCV13 and Hib-MenC observed by the investigator or reported by the participant, will be recorded on the CRF. All solicited and unsolicited adverse events will be recorded for 4 days following immunisation on diary cards. Unsolicited medically significant adverse events occurring between visit 1 and 4 will be recorded on diary cards, as will those occurring between visit 5 and 7.

The following information will be recorded: description, date of onset and end date, severity, assessment of relatedness to study medication, other suspect drug or device and action taken. The relationship of AEs to the study medication will be assessed by a medically qualified investigator.

All SAEs will be recorded through the whole study. Follow-up information should be provided as necessary.

These AEs considered to be related to the study medication as judged by a medically qualified investigator or the sponsor will be followed until resolution or the event is considered stable. All related AEs that result in a participant’s withdrawal from the study or are present at the end of the study, should be followed up until a satisfactory resolution occurs.

It will be left to the investigator’s clinical judgment whether or not an AE is of sufficient severity to require the participant’s removal from the study (see section 6.6). A participant may also be voluntarily withdrawn from the study by a parent/guardian due to what he or she perceives as an intolerable AE. If either of these occurs, the participant must undergo an end of study assessment and be given appropriate care under medical supervision until symptoms cease or the condition becomes stable.

## Reporting Procedures for Serious Adverse Events

The Oxford Radcliffe Hospitals Trust / University of Oxford Trials Safety Group (TSG) will undertake to review immediately reported SAEs for the study.  They will meet at regular intervals and consider:

- Occurrence and nature of adverse events
- Whether additional information on adverse events is required
- Consider taking appropriate action where necessary to halt trials
- Act / advise on incidents occurring between meetings that require rapid assessment (eg SUSARs)

All SAEs (from all sites) must be reported to the University of Oxford CTRG within one working day of discovery or notification of the event. CTRG will perform an initial check of the information and ensure that it is reviewed at the next TSG meeting. All SAE information must be recorded on an SAE form and faxed to CTRG. Additional information received for a case (follow-up or corrections to the original case) need to be detailed on a new SAE form and faxed to CTRG.

The CI will report all SUSARs to the Competent Authorities (MHRA in the UK and MA in Malta) and the Research Ethics Committee concerned. SUSARs occurring in the UK will only be reported to the MHRA, whereas SUSARs occurring in Malta will be reported to the MHRA and the MA. All SUSARs reported to the MHRA and MA will be reported to EudraVigilance. Fatal or life-threatening SUSARs must be reported within 7 days and all other SUSARs within 15 days. The CI will also inform all investigators concerned of relevant information about SUSARs that could adversely affect the safety of participants. The CI will also report SAEs that are considered related to the administration of study vaccines, or to study procedures, in an annual safety report to the Competent Authorities (MHRA in the UK and MA in Malta) and the Research Ethics Committee concerned. All safety reports to the MHRA must be made electronically from the 1^st^ of September 2010.

In addition to the expedited reporting above, the CI shall submit once a year throughout the clinical trial or on request a safety report to the Competent Authority (MHRA in the UK and MA in Malta) and Ethics Committee.

# STATISTICS

## Endpoint analysis will be carried out after all participants have completed visit 7 (13 month blood sample). An additional analysis will be carried out after the final follow-up visit at 24 months of age (visit 8). An interim analysis will be carried out after all participants have completed visit 4 (5 month blood sample).

## 9.1 Description of Statistical Methods

**A)** ***Description of demographics***

Baseline demographic characteristics (age in weeks, gender, race) of each study dose group will be tabulated.

The number of subjects, mean age (plus range and standard deviation) by gender of the enrolled subjects, as a whole, and per study dose group, will be reported.

The trial completion rate will be reported, together with the time and number of withdrawals and the reason for withdrawal, for each study dose group.

**B)** ***Analysis of Immunogenicity***

***Descriptive analysis:***

Summary statistics will be calculated for the following outcomes assessed at 1 month after completion of the primary immunisation, at 12 months plus 6 days and at 12 months plus 28 days and 24 months after the Hib-MenC booster dose for each study dose group. Continuous variables will be reported as means and standard deviations, and categorical variables will be reported as counts and percentages, together with the numbers of observations in all cases.

- MenC rSBA GMT
- Number of MenC specific memory B cells in the blood
- *S. pneumoniae* IgG GMC response for each of the 13 serotypes
- Pneumococcal serotype specific B cell phenotype
- Anti-PRP IgG GMC response
- Anti-tetanus toxoid GMC response
- Percentages of subjects with MenC rSBA ≥1:8 and ≥ 1:128
- Percentages of subjects with MenC rSBA GMT ≥ 1000
- Percentages of subjects with *S. pneumoniae* IgG ≥ 0.35 μg/ml for each of the 13 serotypes
- Percentages of subjects with anti-PRP IgG ≥0.15 μg/ml and ≥ 1.0 μg/ml
- Percentages of subjects with anti-tetanus toxoid > 0.1 IU/ml

***Statistical Analysis***

The analysis of the outcome variables will be performed on the intention–to-treat (ITT) population for the reduced dose MenC objectives and the completers population for the alternating limb objectives. A subject is included in the ITT population if they have at least one dose and at least one post-baseline assessment, and in the completers population if they receive all doses of vaccine and have all planned assessments.

An analysis of variance will be performed when the outcome variable is continuous. The model will contain the terms dose group (4 levels) and alternating limb group (2 levels). A term for centre will be included in the model. The results of a comparison between any two levels of a factor will be reported as a treatment effect with 95% confidence interval.

For the analysis of variables relating only to 0 dose control group the term relating to dose group will be omitted.

The following outcomes will be analysed (comparisons of interest are listed):

1. MenC rSBA GMT response variable assessed 28 days after the Hib-MenC 12 month booster dose
   1. Single dose MenC-CRM_197_ Group vs. Two dose MenC Group
   2. Single dose MenC-CRM_197_ Group vs. 0 dose control Group
   3. Single dose MenC-TT Group vs 0 dose control Group
   4. Two dose MenC Group vs. 0 dose control Group
   5. Single Dose MenC-CRM_197_ Group vs Single Dose MenC-TT Group
2. MenC rSBA GMT response variable assessed 6 days after the Hib-MenC 12 month booster dose
   1. Single dose MenC-CRM_197_ Group vs. Two dose MenC Group
   2. Single dose MenC-CRM_197_ Group vs. 0 dose control Group
   3. Single dose MenC-TT Group vs 0 dose control Group
   4. Two dose MenC Group vs. 0 dose control Group
   5. Single dose MenC-CRM_197_ Group vs Single dose MenC-TT Group

3. MenC rSBA GMT response variable assessed 12 months after the Hib-MenC 12 month booster dose

1. Single dose MenC-CRM_197_ Group vs. Two dose MenC Group
2. Single dose MenC-CRM_197_ Group vs. 0 dose control Group
3. Single dose MenC-TT Group vs 0 dose control Group
4. Two dose MenC Group vs. 0 dose control Group
5. Single dose MenC-CRM_197_ Group vs Single dose MenC-TT Group
6. MenC rSBA GMT response variable (persistence) assessed at 12 months of age
   1. Single dose MenC-CRM_197_ Group vs. Two dose MenC Group
   2. Single dose MenC-CRM_197_ Group vs. 0 dose control Group
   3. Single dose MenC-TT Group vs 0 dose control Group
   4. Two dose MenC Group vs. 0 dose control Group
   5. Single dose MenC-CRM_197_ Group vs Single dose MenC-TT Group
7. MenC rSBA GMT response variable assessed at 5 months of age
   1. Single dose MenC-CRM_197_ Group vs. Two dose MenC Group
   2. Single dose MenC-CRM_197_ Group vs. 0 dose control Group
   3. Single dose MenC-TT Group vs 0 dose control Group
   4. Two dose MenC Group vs. 0 dose control Group
   5. Single dose MenC-CRM_197_ Group vs Single dose MenC-TT Group
8. the numbers of MenC specific memory B cells in the blood at 5 months of age
   1. Single dose MenC-CRM_197_ Group vs. Two dose MenC Group
   2. Single Dose MenC-CRM_197_ Group vs Single Dose MenC-TT Group
9. the numbers of MenC specific memory B cells in the blood at 12 months of age
   1. Single dose MenC-CRM_197_ Group vs. Two dose MenC Group
   2. Single dose MenC-CRM_197_ Group vs. 0 dose control Group
   3. Single dose MenC-TT Group vs 0 dose control Group
   4. Two dose MenC Group vs. 0 dose control Group
   5. Single dose MenC-CRM_197_ Group vs Single dose MenC-TT Group
10. the numbers of MenC specific memory B cells in the blood at 6 days after the Hib-MenC 12 month booster dose
    1. Single dose MenC-CRM_197_ Group vs. Two dose MenC Group
    2. Single dose MenC-CRM_197_ Group vs. 0 dose control Group
    3. Single dose MenC-TT Group vs 0 dose control Group
    4. Two dose MenC Group vs. 0 dose control Group
    5. Single dose MenC-CRM_197_ Group vs Single dose MenC-TT Group
11. The numbers of specific memory B cells in the blood at 28 days after the Hib-MenC 12 month booster dose
    1. Single dose MenC-CRM_197_ Group vs. Two dose MenC Group
    2. Single dose MenC-CRM_197_ Group vs. 0 dose control Group
    3. Single dose MenC-TT Group vs 0 dose control Group
    4. Two dose MenC Group vs. 0 dose control Group
    5. Single dose MenC-CRM_197_ Group vs Single dose MenC-TT Group
12. The numbers of specific memory B cells in the blood at 11-12 months after the Hib-MenC booster dose
    1. Single dose MenC-CRM_197_ Group vs. Two dose MenC Group
    2. Single dose MenC-CRM_197_ Group vs. 0 dose control Group
    3. Single dose MenC-TT Group vs 0 dose control Group
    4. Two dose MenC Group vs. 0 dose control Group
    5. Single dose MenC-CRM_197_ Group vs Single dose MenC-TT Group
13. The *S. pneumoniae* IgG GMC response variable for each of the thirteen serotypes separately at 5 months of age
    1. the consistent limb group vs. the alternating limb group
14. the *S. pneumoniae* IgG GMC response variable for each of the thirteen serotypes separately, (persistence) at 12 months of age
    1. the consistent limb group vs. the alternating limb group

1. the *S. pneumoniae* IgG GMC response variable for each of the thirteen serotypes separately at 13 months of age after the PCV-13 booster dose
   1. the consistent limb group vs. the alternating limb group
2. 12. the *S. pneumoniae* IgG GMC response variable for each of the 13 serotypes separately at 24 months of age.
   1. the consistent limb group vs. the alternating limb group

1. the anti-PRP IgG GMC response variable at 5 months of age
   1. the consistent limb group vs. the alternating limb group
2. the anti-PRP IgG GMC response variable (persistence) at 12 months of age
   1. the consistent limb group vs. the alternating limb group

1. the anti-PRP IgG GMC response variable at 13 months of age, after the Hib-MenC booster dose
   1. the consistent limb group vs. the alternating limb group
2. the anti-PRP IgG GMC response variable at 24 months of age
   1. the consistent limb group vs. the alternating limb group
3. the anti-tetanus toxoid GMC response variable at 5 months of age ,
   1. the consistent limb group vs. the alternating limb group
4. the anti-tetanus toxoid GMC response variable (persistence) at 12 months of age
   1. the consistent limb group vs. the alternating limb group

1. the anti-tetanus toxoid GMC response variable at 13 months of age
   1. the consistent limb group vs. the alternating limb group
2. the anti-tetanus toxoid GMC response variable at 24 months of age
   1. the consistent limb group vs. the alternating limb group

The binary variables will be analysed using logistic regression. The model will contain the terms dose group (4 levels), and alternating limb group (2 levels). There will also be a term for centre. The results of a comparison between two levels of a factor will be reported as an odds ratio and as a risk difference, with 95% confidence intervals.

For the analysis of variables relating only to 0 dose control group the term for dose group will be omitted.

The following binary outcomes will be analysed:

1. percentage of infants with MenC rSBA ≥ 1:8 and ≥ 1:128 assessed 6 days after the 12 month Hib-MenC booster
   1. Single dose MenC-CRM_197_ Group vs. Two dose MenC Group
   2. Single dose MenC-CRM_197_ Group vs. 0 dose control Group
   3. Single dose MenC-TT Group vs 0 dose control Group
   4. Two dose MenC Group vs. 0 dose control Group
   5. Single dose MenC-CRM_197_ Group vs Single dose MenC-TT Group

1. percentage of infants with MenC rSBA ≥ 1:8 and ≥ 1:128 assessed 28 days after the 12 month Hib-MenC booster
   1. Single dose MenC-CRM_197_ Group vs. Two dose MenC Group
   2. Single dose MenC-CRM_197_ Group vs. 0 dose control Group
   3. Single dose MenC-TT Group vs 0 dose control Group
   4. Two dose MenC Group vs. 0 dose control Group
   5. Single dose MenC-CRM_197_ Group vs Single dose MenC-TT Group

1. percentage of infants with MenC rSBA ≥ 1:8 and ≥1:128 assessed at 12 months of age
   1. Single dose MenC-CRM_197_ Group vs. Two dose MenC Group
   2. Single dose MenC-CRM_197_ Group vs. 0 dose control Group
   3. Single dose MenC-TT Group vs 0 dose control Group
   4. Two dose MenC Group vs. 0 dose control Group
   5. Single dose MenC-CRM_197_ Group vs Single dose MenC-TT Group
2. percentage of infants with MenC rSBA ≥ 1:8 and ≥1:128 at 5 months of age
   1. Single dose MenC-CRM_197_ Group vs. Two dose MenC Group
   2. Single dose MenC-CRM_197_ Group vs. 0 dose control Group
   3. Single dose MenC-TT Group vs 0 dose control Group
   4. Two dose MenC Group vs. 0 dose control Group
   5. Single dose MenC-CRM_197_ Group vs Single dose MenC-TT Group
3. percentage of infants with MenC rSBA ≥ 1:8 and ≥1:128 at 24 months of age
   1. Single dose MenC-CRM_197_ Group vs. Two dose MenC Group
   2. Single dose MenC-CRM_197_ Group vs. 0 dose control Group
   3. Single dose MenC-TT Group vs 0 dose control Group
   4. Two dose MenC Group vs. 0 dose control Group
   5. Single dose MenC-CRM_197_ Group vs Single dose MenC-TT Group
4. percentage of infants with MenC rSBA GMT ≥ 1000 assessed 6 days after the 12 month Hib-MenC booster
   1. Single dose MenC-CRM_197_ Group vs. Two dose MenC Group
   2. Single dose MenC-CRM_197_ Group vs. 0 dose control Group
   3. Single dose MenC-TT Group vs 0 dose control Group
   4. Two dose MenC Group vs. 0 dose control Group
   5. Single dose MenC-CRM_197_ Group vs Single dose MenC-TT Group
5. percentage of infants with MenC rSBA GMT ≥ 1000 assessed 28 days after the 12 month Hib-MenC booster
   1. Single dose MenC-CRM_197_ Group vs. Two dose MenC Group
   2. Single dose MenC-CRM_197_ Group vs. 0 dose control Group
   3. Single dose MenC-TT Group vs 0 dose control Group
   4. Two dose MenC Group vs. 0 dose control Group
   5. Single dose MenC-CRM_197_ Group vs Single dose MenC-TT Group

1. percentage of infants with *S. pneumoniae* IgG ≥ 0.35 μg/ml for each of the 13 serotypes at 5 months of age
2. the consistent limb group vs. the alternating limb group
3. percentage of infants with *S. pneumoniae* IgG ≥ 0.35 μg/ml for each of the 13 serotypes at 12 months of age
4. the consistent limb group vs. the alternating limb group
5. percentage of infants with *S. pneumoniae* IgG ≥ 0.35 μg/ml for each of the 13 serotypes at 13 months of age after the PCV-13 booster dose
6. the consistent limb group vs. the alternating limb group
7. percentage of infants with *S. pneumoniae* IgG ≥ 0.35 μg/ml for each of the 13 serotypes at 24 months of age after the PCV-13 booster dose
   1. the consistent limb group vs. the alternating limb group
8. percentage of infants with anti-PRP IgG ≥ 0.15 μg/ml and ≥ 1.0 μg/ml at 5 months of age
9. the consistent limb group vs. the alternating limb group
10. percentage of infants with anti- PRP IgG ≥ 0.15 μg/ml and ≥ 1.0 μg/ml at 12 months of age
11. the consistent limb group vs. the alternating limb group
12. percentage of infants with anti-PRP IgG ≥ 0.15 μg/ml and ≥ 1.0 μg/ml at 13 months of age, after the Hib-MenC booster dose
    1. the consistent limb group vs. the alternating limb group
13. percentage of infants with anti-PRP IgG ≥ 0.15 μg/ml and ≥ 1.0 μg/ml at 24 months of age
    1. the consistent limb group vs. the alternating limb group
14. percentage of infants with anti- tetanus toxoid >0.1 IU/ml at 5 months of age
15. the consistent limb group vs. the alternating limb group
16. percentage of infants with anti- tetanus toxoid >0.1 IU/ml at 12 months of age
17. the consistent limb group vs. the alternating limb group
18. percentage of infants with anti- tetanus toxoid >0.1 lU/ml at 13 months of age
    1. the consistent limb group vs. the alternating limb group
19. percentage of infants with anti- tetanus toxoid >0.1 lU/ml at 24 months of age
    1. the consistent limb group vs. the alternating limb group

**C) Analysis of safety**

The data summaries will report the total number and percentage of infants experiencing each type of adverse event, and the total number and percentage of infants experiencing at least one adverse event of any type, for each dose group, and the total number of infants in each dose group, for local and general adverse events during the 4-day follow-up period after each MenC (including Hib-MenC), DTaP-IPV-Hib and PCV13 vaccination. The classification of severity of local adverse events and fever will follow the Brighton collaboration guidelines ^35 - 38^.

Where the adverse event is further classified with a grade (1, 2, 3) related to severity, the summary reports will include the numbers in each class.

**Statistical analysis**

The binary variables will be analysed using logistic regression. The model will contain the terms dose group (4 levels) and alternating limb group (2 levels). A term for centre will be included in the model. The results of a comparison between two levels of a factor will be reported as an odds ratio and as a risk difference, with 95% confidence intervals.

The following variables will be analysed:

**Local adverse events**

1. percentage of infants with each type of adverse event, and at least one local adverse event after each dose of MenC vaccine (at 3 and 4 months)
   1. Single dose MenC-CRM_197_ Group vs. Two dose MenC Group
   2. Single Dose MenC-CRM_197_ Group vs Single Dose MenC-TT Group
2. percentage of infants with each type of adverse event, and at least one local adverse event after each dose of DTaP-IPV-Hib (at 2, 3 and 4 months) and PCV13 (at 2 and 4 months)
   1. the consistent limb group vs. the alternating limb group
3. percentage of infants with each type of adverse event, and at least one local adverse event after the 12 month booster Hib-MenC and PCV13 vaccination
   1. Two dose MenC Group vs. 0 dose control Group
   2. Single dose MenC-CRM_197_ Group vs. 0 dose control Group
   3. Single dose MenC-CRM_197_ Group vs. Two dose MenC Group
   4. Single dose MenC-TT Group vs 0 dose control Group
   5. Single dose MenC-CRM_197_ Group vs Single dose MenC-TT Group
   6. the consistent limb group vs. the alternating limb group

**Systemic adverse events**

1. percentage of infants with each type of adverse event, and at least one systemic adverse event after MenC (3 and 4 months), DTaP-IPV-Hib (2, 3 and 4 months) and PCV13 (2 and 4 months) vaccination
   1. Two dose MenC Group vs. 0 dose control Group
   2. Single dose MenC-CRM_197_ Group vs. 0 dose control Group
   3. Single dose MenC-CRM_197_ Group vs. Two dose MenC Group
   4. Single dose MenC-TT Group vs 0 dose control Group
   5. Single dose MenC-CRM_197_ Group vs Single dose MenC-TT Group
   6. the consistent limb group vs. the alternating limb group
2. percentage of infants with each type of adverse event, and at least systemic one adverse event after the 12 month booster Hib-MenC and PCV13 vaccination
   1. Two dose MenC Group vs. 0 dose control Group
   2. Single dose MenC-CRM_197_ Group vs. 0 dose control Group
   3. Single dose MenC-CRM_197_ Group vs. Two dose MenC Group
   4. Single dose MenC-TT Group vs 0 dose control Group
   5. Single dose MenC-CRM_197_ Group vs Single dose MenC-TT Group
   6. The consistent limb group vs. the alternating limb group

## 9.2 The Number of Participants

This is a non-inferiority trial assessing whether there is any difference in the antibody level between infants who receive no priming doses of MenC and those who receive 1 priming dose or 2 priming doses of MenC-CRM_197_.

The total sample size for the study is 498 participants randomised on a 10:10:7:4 basis to the 4 study groups. This will result in 160 participants in the Two Dose MenC group, 160 in the Single Dose MenC-CRM_197_ group, 114 in the Single Dose MenC-TT group, and 64 in 0 dose MenC (Control) group. Based on a mean (SD) log SBA of 3.47 (0.9) at 1 month after booster for 2 dose MenC^12^, 160 participants per group are required for the comparison between the Single Dose MenC-CRM_197_ and the Two dose MenC groups to detect 10% non-inferiority with a 2.5% level of significance (1-sided), 90% power and allowing for a 12.5% drop-out rate. An additional 114 participants will be required for the Single Dose MenC-TT group to allow a comparison with the Single Dose MenC-CRM_197_ primed group, allowing for a 12.5% drop-out rate.

No sample size calculation for the alternate limb component of the trial is included as the data generated here will be pilot data.

| Non-inferiority margi | Estimated difference | Power | Sample size required per group | Total sample size required |
| --- | --- | --- | --- | --- |
| 5% | -0.18 | 90% | 526 | 1052 |
|  |  | 80% | 393 | 786 |
| 7.5% | -0.26 | 90% | 253 | 506 |
|  |  | 80% | 190 | 380 |
| **10%** | **-0.35** | **90%** | **140*** | **280** |
|  |  | 80% | 105 | 210 |

** Requires 160 participants per group when allowing for a ‘dropout’ rate of 12.5%*

A previous study has shown the mean (SD) logSBA=2.85 (0.48) at 1 month following a 12 month dose of MenC vaccine with no prior MenC immmunisation^43^. Making the reasonable assumption that the SBA titres are relatively stable in the one month period after the vaccination, these statistics were used to calculate a sample size for a comparison between the one dose group and 0 dose control group at 6 days after the 12 month vaccination.

To detect a difference of 0.285 (10% of 2.85) between groups of equal size of 56 with a significance level of 0.05 (2-sided), would provide a power of 88%. Allowing for 12.5% drop outs, the initial size of 0 dose control group should be 64 infants. This therefore enables the 6 day blood test to be performed on only a subset of participants (64) in each of the other groups.

***Assumptions***

Sample size calculation has been extrapolated from the following MenC rSBA data taken from the infant MenC study^39^, at various time points:

12 months

Log10 rSBA : mean= 0.9441395 Std. Dev = 0.8876458

Log10 IgG : mean= 0.02791 Std. Dev = 0.4380407

6 days-post 12 months booster

Log10 rSBA : mean= 3.672566 Std. Dev = 0.2518598

Log10 IgG : mean= 1.211286 Std. Dev.= 0.1606558

8 days-post 12 months booster

Log10 rSBA : mean= 4.264592 Std. Dev = 0.584236

Log10 IgG : mean= 1.72307 Std. Dev.= 0.3447821

30 days-post 12 months booster

Log10 rSBA: mean= 3.784377 Std. Dev = 0.4211916

Log10 IgG: mean= 1.446253 Std. Dev.= 0.3564013

## 9.3 Hypothesis Test

Non-inferiority of Single Dose MenC-CRM_197_ schedule over Two Dose MenC schedule would be demonstrated if the null hypothesis bellow is rejected.

**Null Hypothesis H0**:

The mean of (log_10_) of MenC rSBA values of the active current vaccine schedule (Two Dose) group μ_exp_, exceeds the mean of (log_10_) of MenC rSBA values of the experimental vaccine schedule (Single Dose) group μ_ac_ by at least a margin “M” (M >0).

**H_0_**: μ_exp_ –μ_ac_ ≤ -M

**Alternative Hypothesis H_a_**: The mean of (log_10_) of MenC rSBA values of the active current vaccine schedule (Two Dose) may indeed have higher mean of (log_10_) of MenC rSBA values compared to mean of (log_10_) of MenC rSBA of the experimental vaccine schedule (Single Dose) group, but the difference is not more than “M” (M >0).

**Ha**: μ_exp_ –μ_ac_ > -M

- μ_exp_ refers to the population mean of log_10_ of MenC rSBA values for the experimental vaccine schedule (Single dose) group
- μ_ac_ refers to the population mean of log_10_ of MenC rSBA values of the active current vaccine schedule (Two Dose) group.

For the above comparison, M, the non-inferiority margin, is chosen to be 0.35, this value corresponds to a 10% of the value 3.47 log_10_ (MenC rSBAGMT) at 1 month after booster (Reference 14, Borrow et al)

μ_exp_ and μ_ac_ can be estimated as log_10_ (MenC rSBAGMT) in (Single Dose) and (Two Dose) groups respectively (which are equivalent to the arithmetic mean of log_10_ MenCrSBA in (Single Dose) group and the arithmetic mean of log_10_ MenCrSBA in (Two Dose) group).

The nominal significance level for this one-sided test is considered α=0.025 and power is 90%.

If the null hypothesis is rejected we can then conclude non-inferiority of the experimental Single Dose priming vaccine schedule compared to Two Dose priming vaccine schedule.

## 9.4 Criteria for the Termination of the Trial.

The end of trial is the date of the completion of all study assays. This is a Phase IV trial so it is not expected that the clinical trial would terminated prematurely because of adverse effects on the participants. It is possible, however, that an increase in the incidence of serogroup C meningococcal disease could change the risk/ benefit profile of participants (particularly those in the 0 dose MenC (Control) group). No such increase in MenC disease is anticipated, however if this were to occur the appropriateness or otherwise of continuing the study will be considered by the data monitoring committee and trial steering committee.

## 9.5 Procedure for Accounting for Missing, Unused, and Spurious Data.

Missing data will not be accounted for.

## 9.6 Procedures for Reporting any Deviation(s) from the Original Statistical Plan

Any significant deviation from the original statistical plan will be discussed with the study statistician and, as appropriate, reflected in any publications arising out of this study.

## 9.7 Inclusion in Analysis

The immunogenicity analysis will be performed on both an intention to treat (ITT) population (all participants completing their designated primary and booster stage immunisation courses, and providing blood samples at the timepoint being analysed) and on a completers protocol (CP) population (all participants in the intention to treat analysis completing the study without any significant protocol deviations).

# Direct Access to Source Data/Documents

Direct access will be granted to authorised representatives from the sponsor, host institution and the regulatory authorities to permit trial-related monitoring, audits and inspections.

# Quality Control and Quality Assurance Procedures

The study will be conducted in accordance with the current approved protocol, ICH GCP, relevant regulations and standard operating procedures.

Regular monitoring will be performed according to ICH GCP. Data will be evaluated for compliance with the protocol and accuracy in relation to source documents. A risk based monitoring plan will be developed and used by the monitors to verify that the clinical trial is conducted and data are generated, documented and reported in compliance with the protocol, relevant standard operating procedures, GCP and all applicable regulatory requirements.

A trial steering committee will be formed that will include, but not be limited to, the chief investigator, a statistician, a quality assurance manager and project manager.

# Ethics

This study will recruit infants who are not old enough to give consent. Informed consent will be obtained from the parent/legal guardian of each participant.

## 12.1 Declaration of Helsinki

The Investigator will ensure that this study is conducted in accordance with the principles of the Declaration of Helsinki.

## 12.2 ICH Guidelines for Good Clinical Practice

The Investigator will ensure that this study is conducted in full conformity with relevant regulations and with the ICH Guidelines for Good Clinical Practice (CPMP/ICH/135/95) July 1996.

## 12.3 Approvals

The protocol, informed consent form, participant information sheet and any proposed advertising material will be submitted to the Research Ethics Committee (REC) and a favourable opinion will be sought in both countries. Approval from the MHRA and from the relevant Research and Development departments in the Primary Care Trusts where the study will be conducted will be obtained prior to starting the study in the UK. In Malta approval from the MA and from the Chairman of Paediatrics and the Hospital’s Superintendent at Mater Dei Hospital will be obtained prior to starting the study. The Investigator will submit and, where necessary, obtain approval from the above parties for all substantial amendments to the original approved documents.

## 12.4 Participant Confidentiality

The trial staff will ensure that the participants’ anonymity is maintained. The participants will be identified only by initials and a participant number on the CRF and any electronic database. All documents will be stored securely and only accessible by trial staff and authorised personnel. The study will comply with the Data Protection Act which requires data to be anonymised as soon as it is practical to do so. Given the possibility of enrolling twins into this study the study diaries will be labelled with the participant’s first name to aid recording of data relevant to the appropriate child.

**12.5 Other Ethical Considerations**

Participants might sustain pain at the site of venepuncture. Pain will be reduced by applying a local anaesthetic cream which will temporarily desensitise the skin over the venepuncture site. Up to two attempts may be made to obtain a blood sample at each visit, if the parent gives verbal consent at the time of the procedure for a second attempt. If no blood is obtained, a second visit may be made only if the parent agrees to this.

Pain associated with immunisation will be reduced by allowing the infant to breastfeed or by using distraction methods.

If a participant was found to have antibody levels below the threshold of response for any of the vaccines received in this study on the 24 month blood sample (or after the 13 month blood sample if the participant did not return for the final study visit), then study staff would administer a booster dose of the relevant vaccine at a later visit to be organised with the participant’s family. This booster may be administered by the participant’s GP if necessary (for example if the participant has moved out of the area) and if this agreed with the participant’s family and GP.

# Data Handling and Record Keeping

All study data will be entered into a sharepoint based database system.

The participants will be identified by a study specific participant number and/or initial in any database. The name and any other identifying detail will NOT be included in any study data electronic file.

# Financing and Insurance

Part financing has been obtained from the BRC feasibility and sustainability fund. Insurance will be provided by the University of Oxford in the UK.

# Publication Policy

Publications arising from this paper will be coordinated by the chief investigator.

# Storage and handling of samples

Samples will not be labelled with information that directly identifies the subjects but will be coded with the participant number/initials for the subject. After blood centrifugation and serum separation, samples will be stored at -20°C until analysis can be performed. Storage and handling of biological samples will be according to the site specific Laboratory Standard Operating Procedures (SOPs).

B cell responses will be determined in participants enrolled at Oxford and, potentially, other study sites according to local arrangements. At Oxford this analysis will be conducted on fresh samples. For samples obtained elsewhere the analysis will be conducted either at Oxford on frozen samples transferred from the other study sites or analysed locally on fresh or frozen samples. Where applicable, antigen specific B cell responses to vaccines received in the study will be analysed in the Oxford Vaccine Centre laboratory at the Centre for Clinical Vaccinology and Tropical Medicine, University of Oxford by ELISPOT and flow cytometric assays depending on the available volumes of blood.

DNA will be extracted from a sample of blood according to standard protocol. The cellular plug resting after centrifugation of the sample of blood may be kept in -20° C for later DNA extraction. These blood clots and the extracted DNA will be stored in the Biobank of the Oxford Vaccine Centre laboratory at the Centre of Clinical Vaccinology and Tropical Medicine, University of Oxford or at local study sites for later analysis for genetic polymorphisms. Genetic testing will be performed on these samples before January 2015.

# REFERENCES

1. Bilukha OO, Rosenstein N, National Center for Infectious Diseases, Centers for Disease Control and Prevention (CDC). Prevention and control of meningococcal disease. Recommendations of the Advisory Committee on Immunization Practices (ACIP). MMWR Recomm Rep. 2005 May 27; 54(RR-7):1-21.
2. European Union Invasive Bacterial Infections Surveillance Network. 2003 Invasive *Neisseira meningitidis* in Europe-2002. Available from www.euibis.org/documents/2002_meningo.pdf.
3. Whalen CM, Hockin JC, Ryan A et al. The changing epidemiology of invasive meningococcal disease in Canada, 1985 through 1992: emergence of a virulent clone of Neisseria meningitidis. *JAMA.* 1995;273:390-394.
4. Pollard AJ, Levin M. Vaccines for prevention of meningococcal disease. Pediatr Infect Dis J. 2000; 19(4): 333-44.
5. Environmental Science and Research Limited. 2006. The Epidemiology of Meningococcal Disease in New Zealand in 2006. Wellington: Ministry of Health. Available from http://www.moh.govt.nz/moh.nsf/pagesmh/6647 /$File/epidemiology-of-meningococcal-disease-2006.pdf
6. Rosenstein NE, Perkins BA, Stephens DS, Popovic T, Hughes JM. Meningococcal disease. N Engl J Med. 2001 May 3;344(18):1378-88.
7. Snape MD, Pollard AJ. Meningococcal polysaccharide-protein conjugate vaccines. Lancet Infect Dis. 2005; 5(1): 21-30.
8. Granoff DM, Pollard AJ. Reconsideration of the use of meningococcal polysaccharide vaccine. Pediatr Infect Dis J. 2007 Aug;26(8):716-22.
9. Schittek B, Rajewsky K. Maintenance of B-cell memory by long-lived cells generated from proliferating precursors. Nature. 1990 Aug 23;346(6286):749-51.
10. MacLennan IC, Gulbranson-Judge A, Toellner KM, Casamayor-Palleja M, Chan E, Sze DM, et al. The changing preference of T and B cells for partners as T-dependent antibody responses develop. Immunol Rev. 1997 Apr;156:53-66.
11. Pace D, Cuschieri P, Galea Debono A, Attard-Montalto S. [Epidemiology of pathogenic Neisseria meningitidis serogroup B serosubtypes in Malta: implications for introducing PorA based vaccines.](http://www.ncbi.nlm.nih.gov/pubmed/18801401?itool=EntrezSystem2.PEntrez.Pubmed.Pubmed_ResultsPanel.Pubmed_RVDocSum&ordinalpos=12) Vaccine. 2008 Nov 5;26(47):5952-6.
12. Muscat M, Spiteri G, Calleja N, Haider J, Gray SJ, Melillo JM, et al. I[nvasive meningococcal disease in Malta: an epidemiological overview, 1994-2007.](http://www.ncbi.nlm.nih.gov/pubmed/19589903?itool=EntrezSystem2.PEntrez.Pubmed.Pubmed_ResultsPanel.Pubmed_RVDocSum&ordinalpos=3) J Med Microbiol. 2009 Nov;58(Pt 11):1492-8.
13. Miller E, Pollard AJ, Borrow R, et al. Safety and immunogenicity of Novartis meningococcal serogroup B vaccine after three doses administered in infancy. Paper presented at: ESPID 2008; Graz.
14. Borrow R, Goldblatt D, Finn A, et al. Immunogenicity of, and immunologic memory to, a reduced primary schedule of meningococcal C-tetanus toxoid conjugate vaccine in infants in the United Kingdom. Infect Immun. Oct 2003;71(10):5549-5555.
15. Southern J, Crowley-Luke A, Borrow R, Andrews N, Miller E. Immunogenicity of one, two or three doses of a meningococcal C conjugate vaccine conjugated to tetanus toxoid, given as a three-dose primary vaccination course in UK infants at 2, 3 and 4 months of age with acellular pertussis-containing DTP/Hib vaccine. Vaccine*.* Jan 12 2006;24(2):215-219.
16. Richmond P, Borrow R, Miller E, et al. Meningococcal serogroup C conjugate vaccine is immunogenic in infancy and primes for memory. J Infect Dis. Jun 1999;179(6):1569-1572.
17. Richmond P, Borrow R, Findlow J, et al. Evaluation of De-O-acetylated meningococcal C polysaccharide-tetanus toxoid conjugate vaccine in infancy: reactogenicity, immunogenicity, immunologic priming, and bactericidal activity against O-acetylated and De-O-acetylated serogroup C strains. Infect Immun. 2001;69:2378-2382.
18. Snape MD, Kelly DF, Lewis S, Banner C, Kibwana L, Moore CE, et al. Seroprotection against serogroup C meningococcal disease in adolescents in the United Kingdom: observational study. BMJ. 2008 Jun 28;336(7659):1487-91.
19. Miller E, Salisbury D, Ramsay ME. Planning, registration and implementation of an immunisation campaign against meningococcal serogroup C disease in the UK: a success story. Vaccine 2001; 20 Suppl 1: S58-67.
20. Trotter C, Andrew N, Kaczmarski E, Miller E, Ramsay M. Effectiveness of meningococcal serogroup C conjugate vaccine 4 years after introduction. Lancet. 2004; 364 (9431): 365-367.
21. Maiden MC, Stuart JM. Carriage of serogroup C meningococci 1 year after meningococcal C conjugate polysaccharide vaccination. *Lancet.* May 25 2002;359(9320):1829-1831.
22. A Phase IV, Randomized Study to Evaluate the Immune Response of UK Infants Receiving DTaP/Hib/IPV, Meningococcal C Conjugate and Pneumococcal Conjugate Vaccines, Antibody Persistence and Responses to Booster Doses in the Second Year of Life (Sched2). http://clinicaltrials.gov/ct2/show/NCT00625677? term=sched2&rank=1. Accessed 15th November, 2008.
23. Borrow R, Andrews N, Findlow H, Waight P, Southern J, Crowley-Luke A, Stapley L, England A, Findlow J, Miller E. [Kinetics of antibody persistence following administration of a combination meningococcal serogroup C and haemophilus influenzae type b conjugate vaccine in healthy infants in the United Kingdom primed with a monovalent meningococcal serogroup C vaccine.](http://www.ncbi.nlm.nih.gov/pubmed/19906895?itool=EntrezSystem2.PEntrez.Pubmed.Pubmed_ResultsPanel.Pubmed_RVDocSum&ordinalpos=1) Clin Vaccine Immunol 2010; 17: 154-9.
24. *Recommendations for the production and control of meningococcal group C conjugate vaccines*: World Health Organisation; 2004. 924.
25. Pollard AJ, Granoff DM. Reconsideration of the Use of Meningococcal Polysaccharide Vaccine. *Paed Infect Dis J.* 2007;26:716 - 722.
26. World Health Authority ECoBS. Proposed replacement of: TRS 926, Annex 3. Part C. Clinical evaluation of group C meningococcal conjugate vaccines. Accessed 28 September, 2007. http://www.who.int/biologicals/expert_committee/BS2065%20Mening%20+%20line%20number.pdf.
27. Ponzio NM, Chapman-Alexander JM, Thorbecke GJ. Transfer of memory cells into antigen-pretreated hosts. I. Functional detection of migration sites for antigen-specific B cells. Cell Immunol. 1977 Nov;34(1):79-92.
28. Peck FB Jr, Kohlstaedt KC. Pre-exposure rabies prophylaxis problems and procedures. Ind Med Surg. 1964 Jan;33:17-21.
29. Tan PL, Jacobson RM, Poland GA, Jacobsen SJ, Pankratz VS. Twin studies of

immunogenicity--determining the genetic contribution to vaccine failure. Vaccine

2001; 19(17-19): 2434–9.

1. Newport MJ, Goetghebuer T, Weiss HA, Whittle H, Siegrist CA, Marchant A.

Genetic regulation of immune responses to vaccines in early life. Genes Immun 2004;5:122–9.

1. Ovsyannikova IG, Pankratz VS, Vierkant RA, Jacobson RM, Poland GA. Human

leukocyte antigen haplotypes in the genetic control of immune response to measles mumps-rubella vaccine. J Infect Dis 2006; 19:655-63.

1. Yucesoy B, Sleijffers A, Kashon M, Garssen J, de Gruijl FR, Boland GJ, et al. IL-1beta gene polymorphisms influence hepatitis B vaccination. Vaccine 2002; 20: 3193-6.
2. Summary of product characteristics; Menjugate Kit. Novartis Vaccines and Diagnostics. Available at http://emc.medicines.org.uk
3. Summary of product characteristics; Pediacel, Sanofi Pasteur, MSD. Available at [http://www.medicines.org.uk](http://www.medicines.org.uk/)
4. Summary of product characteristics; NeisVac-C. Baxter Vaccines. Available at http://www.medicines.org.uk
5. Summary of product characteristics; Prevenar-13, Wyeth Lederle Vaccines. Available at [http://www](http://www/).medicines.org.uk
6. Summary of product characteristics; Menitorix, GlaxoSmithKline. Available at [http://www](http://www/).medicines.org.uk
7. UK Department of Health. Immunisation against infectious diseases. Green book, 2006. Chapter 4: Immunisation procedures.
8. Michael Marcy S, Kohl KS, Dagan R, Nalin D, Blum M, Jones MC, et al. Brighton Collaboration Fever Working Group. Fever as an adverse event following immunization: case definition and guidelines of data collection, analysis, and presentation. Vaccine. 2004 Jan 26;22(5-6):551-6.
9. Kohl KS, Walop W, Gidudu J, Ball L, Halperin S, Hammer SJ, et al. Brighton Collaboration Local Reactions Working Group for Induration at or near Injection Site. Induration at or near injection site: case definition and guidelines for collection, analysis, and presentation of immunization safety data. Vaccine. 2007 Aug 1;25(31):5839-57.
10. Kohl KS, Walop W, Gidudu J, Ball L, Halperin S, Hammer SJ, et al. Brighton Collaboration Local Reaction Working Group for Swelling at or near Injection Site. Swelling at or near injection site: case definition and guidelines for collection, analysis and presentation of immunization safety data. Vaccine. 2007 Aug 1;25(31):5858-74.
11. Gidudu J, Kohl KS, Halperin S, Hammer SJ, Heath PT, Hennig R, et al. Brighton Collaboration Local Reactions Working Group for a Local Reaction at or near Injection Site. A local reaction at or near injection site: case definition and guidelines for collection, analysis, and presentation of immunization safety data. Vaccine. 2008 Dec 9;26(52):6800-13.
12. Snape MD, MacLennan J, Lockhart S, et al. Demonstration of immunologic memory using serogroup C meningococcal glyco-conjugate vaccine. *Paed Infect Dis J.* 2009. Feb;28(2):92-7.

# APPendix a: study flow CharT

| **Details of vaccine administration for each treatment group** | | | **Visit 1** | **Visit 2** | **Visit 3** | **Visit 4** | **Visit 5** | **Visit 6*** | **Visit 7** | **Visit 8** |
| --- | --- | --- | --- | --- | --- | --- | --- | --- | --- | --- |
|  |  |  |  |  |  | **Blood** | **Blood** | **Blood** | **Blood** | **Blood** |
| **Two Dose MenC Group**  **(2 doses MenC-CRM_197_)**  **Group 2** | **a** | Right leg | DTaP-IPV-Hib  PCV13 | DTaP-IPV-Hib | DTaP-IPV-Hib  PCV13 |  | PCV13 |  | MMR |  |
|  |  | Left leg |  | *MenC****-CRM_197_*** | *MenC****-CRM_197_*** |  | Hib-MenC |  |  |  |
|  | **b** | Right leg |  | DTaP-IPV-Hib | DTaP-IPV-Hib  PCV13 |  |  |  | MMR |  |
|  |  | Left leg/ arm | DTaP-IPV-Hib  PCV13 | *MenC***-*CRM_197_*** | *MenC****-CRM_197_*** |  | Hib-MenC (leg), PCV13 (arm) |  |  |  |
| **Single Dose MenC-CRM_197_ Group**  **(1 dose MenC-CRM_197_)**  **Group 1** | **a** | Right leg | DTaP-IPV-Hib  PCV13 | DTaP-IPV-Hib | DTaP-IPV-Hib  PCV13 |  | PCV13 |  | MMR |  |
|  |  | Left leg/ arm |  | *MenC***-*CRM_197_*** |  |  | Hib-MenC |  |  |  |
|  | **b** | Right leg |  | DTaP-IPV-Hib | DTaP-IPV-Hib  PCV13 |  |  |  | MMR |  |
|  |  | Left leg/ arm | DTaP-IPV-Hib  PCV13 | *MenC****-CRM_197_*** |  |  | Hib-MenC (leg) PCV13 (arm) |  |  |  |
| **Control Group**  **(0 dose MenC priming)**  **Group 3** | **a** | Right leg | DTaP-IPV-Hib  PCV13 | DTaP-IPV-Hib | DTaP-IPV-Hib  PCV13 |  | PCV13 |  | MMR |  |
|  |  | Left leg/ arm |  |  |  |  | Hib-MenC |  |  |  |
|  | **b** | Right leg |  | DTaP-IPV-Hib | DTaP-IPV-Hib  PCV13 |  |  |  | MMR |  |
|  |  | Left leg/ arm | DTaP-IPV-Hib  PCV13 |  |  |  | Hib-MenC (leg) PCV13 (arm) |  |  |  |
| **Single Dose MenC-TT Group**  **(1 dose MenC-TT)**  **Group 4** | **a** | Right leg | DTaP-IPV-Hib  PCV13 | DTaP-IPV-Hib | DTaP-IPV-Hib  PCV13 |  | PCV13 |  | MMR |  |
|  |  | Left leg/ arm |  | *MenC-TT* |  |  | Hib-MenC |  |  |  |
|  | **b** | Right leg |  | DTaP-IPV-Hib | DTaP-IPV-Hib  PCV13 |  |  |  | MMR |  |
|  |  | Left leg/ arm | DTaP-IPV-Hib  PCV13 | *MenC-TT* |  |  | Hib-MenC (leg) PCV13 (arm) |  |  |  |

* This would be for all participants in the Control Group (ie 64), and a matching number of participants in the Two Dose MenC, Single Dose MenC-CRM_197_ and the Single Dose MenC-CRM_197_ groups as determined by randomisation at enrolment. Timelines: Visit 1: age 6-12 weeks, Visit 2: age approximately 3 months, Visit 3: age approximately 4 months, Visit 4: age approximately 5 months, Visit 5: age approximately 12 months, Visit 6:6 days after visit 5, Visit 7: age approximately 13 months, Visit 8: age approximately 24 months

# APPENDIX B: SCHEDULE OF PROCEDURES

| **Visit** | Visit 1 | Visit 2 | Visit 3 | Visit 4 | Visit 5 | Visit 6* | Visit 7 | Visit 8 |
| --- | --- | --- | --- | --- | --- | --- | --- | --- |
| Informed consent | X |  |  |  |  |  |  |  |
| Check inclusion criteria | X |  |  |  |  |  |  |  |
| Check exclusion criteria | X |  |  |  |  |  |  |  |
| Check elimination criteria |  | X | X | X | X | X | X | X |
| Check contraindications |  | X | X |  | X |  | X |  |
| Medical history | X |  |  |  |  |  |  |  |
| Vaccination history | X |  |  |  |  |  |  |  |
| Pre-vaccination body temperature | X | X | X |  | X |  | X |  |
| Randomisation | X |  |  |  |  |  |  |  |
| Blood sampling: for antibody and B memory cell determination (5ml at 5 months, 7.5ml for older ages) |  |  |  | X | X | X | X | X |
| Vaccination** | X | X | X |  | X |  | X |  |
| Daily post-vaccination recording of solicited symptoms (Days 0–3) by subjects’ parents/guardians | X | X | X |  | X |  |  |  |
| Return of diary cards |  | x | x | x |  | x |  |  |
| Diary card transcription |  | x | x | x |  | x |  |  |
| Record any concomitant immunosuppressive medication/ vaccination | X | X | X | X | X | X | X | X |
| Reporting of Serious Adverse Events |  | X | X | X | X | X | X | X |
| Conclusion of study |  |  |  |  |  |  |  | x |

* This would be for all participants in the Control Group (ie 64), and a matching number of participants in the Two Dose MenC, Single Dose MenC-CRM_197_ and the Single Dose MenC-CRM_197_ groups, as determined by randomisation at enrolment. Timelines: Visit 1: age 6-12 weeks, Visit 2: age approximately 3 months, Visit 3: age approximately 4 months, Visit 4: age approximately 5 months, Visit 5: age approximately 12 months, Visit 6:6 days after visit 5, Visit 7: age approximately 13 months, Visit 8: age approximately 24 months

** Vaccination as per study flow chart in Appendix A.

# 20 Appendix C: Intervals between Visits

Primary phase (initial visit at 6 to 12 weeks of age)

| **Interval** | **Length of interval** |
| --- | --- |
| 1 (Visit 1 to Visit 2) | 28-42 days |
| 2 (Visit 2 to Visit 3) | 28-42 days |
| 3 (Visit 3 to Visit 4) | 28-42 days |

Booster Phase (initial visit at 12 to 13 months of age)

| 5 (Visit 5 to Visit 6) | 6 days |
| --- | --- |
| 6 (Visit 5 to visit 7) | 28 – 42 days |
| 7 (Visit 5 to visit 8) | 11-12 months |
