# Supplementary material for: Evaluation of the Induction of Immune Memory following Infant Immunisation with Serogroup C Neisseria meningitidis Conjugate Vaccines – Exploratory Analyses within a Randomised Controlled Trial
Source: PLoS One. 2014 Jul 14;9(7):e101672. doi: 10.1371/journal.pone.0101672 (PMC4096514; doi:10.1371/journal.pone.0101672)
Supplement: File S1 — Supplemental data tables: Table S1: Comparison of the log10 transformed number of MenC-specific memory B-cells detected in the peripheral blood for each study group against the control group at visits 4, 5 and 6 (5 months, 12 months and 12 months +6 days). Table S2: Differences in log10 transformed number of MenC-specific memory B-cells detected in the peripheral blood between 13-month and 12-month samples for each study group. Table S3: Differences in the log10 transformed number of antigen-specific memory B-cells detected in the peripheral blood between 5 months and 12 months for each study group. (DOCX) [file pone.0101672.s003.docx]

**Supplemental data table S1: Comparison of the log_10_ transformed number of MenC-specific memory B-cells detected in the peripheral blood for each study group against the control group at visits 4, 5 and 6 (5 months, 12 months and 12 months + 6 days).**

|  | | *Kruskal-Wallis statistic* | *p-value* |
| --- | --- | --- | --- |
| **5 months of age (1 month after primary vaccines)** | | | |
| Groups 1-dose CRM, 2-dose CRM, 1-dose TT vs control | | Χ^2^_1_ = 10.2 | **0.001** |
| 1-dose CRM vs control | | Χ^2^_1_ = 4.3 | **0.04** |
| 2-dose CRM vs control | | Χ^2^_1_ = 11.7 | **0.0006** |
| 1-dose TT vs control | | Χ^2^_1_ = 10.6 | **0.001** |
|  | *F test and unadjusted p-value* | *Difference between groups with estimated 95% CI* | *Bonferroni adjusted*  *p-value* |
| **12 months of age (pre-booster)** | | | |
| Groups 1-dose CRM, 2-dose CRM, 1-dose TT vs control | F_1, 169_ = 16.6, p = 0.0001 |  |  |
| 1-dose CRM vs control | F_1, 169_ = 20.3, p <0.0001 | 0.58 (0.33 to 0.84) | **<0.0001** |
| 2-dose CRM vs control | F_1, 169_ = 8.0, p =0.005 | 0.39 (0.12 to 0.67) | **0.03** |
| 1-dose TT vs control | F_1, 169_ = 11.2, p =0.001 | 0.48 (0.20 to 0.76) | **0.006** |
| **12 months + 6 days (6 days after booster vaccination)** | | | |
| Groups 1-dose CRM, 2-dose CRM, 1-dose TT vs control | F_1, 72_ = 49.9 p <0.0001 |  |  |
| 1-dose CRM vs control | F_1, 72_ =40.7, p <0.00001 | 1.09 (0.75 to 1.43) | **<0.0001** |
| 2-dose CRM vs control | F_1, 72_ = 23.5, p <0.00001 | 0.87 (0.51 to 1.22) | **<0.0001** |
| 1-dose TT vs control | F_1, 72_ = 36.4, p <0.00001 | 1.15 (0.77 to 1.53) | **<0.0001** |

1-dose CRM: 1 dose MenC-CRM_197_ at 3 months of age; 2-dose CRM: 2 doses of MenC-CRM_197_ at 3 and 4 months of age; Control: No MenC primary vaccine doses; 1-dose TT: 1 dose MenC-TT at 3 months of age

**Supplemental data table S2:** **Differences in log_10_ transformed number of MenC-specific memory B-cells detected in the peripheral blood between 13-month and 12-month samples for each study group.**

| **Group** | **Mean difference** | **Standard error** | **Estimated 95% CI around difference** | **p-value** |
| --- | --- | --- | --- | --- |
| 1-dose CRM | 0.222 | 0.104 | 0.02 to 0.42 | **0.03** |
| 2-dose CRM | 0.326 | 0.119 | 0.09 to 0.56 | **0.006** |
| Control | 0.911 | 0.166 | 0.58 to 1.24 | **<0.0001** |
| 1-dose TT | 0.829 | 0.137 | 0.56 to 1.10 | **<0.0001** |

1-dose CRM: 1 dose MenC-CRM_197_ at 3 months of age; 2-dose CRM: 2 doses of MenC-CRM_197_ at 3 and 4 months of age; Control: No MenC primary vaccine doses; 1-dose TT: 1 dose MenC-TT at 3 months of age

**Supplemental data table S3: Differences in the** **log_10_ transformed number of antigen-specific memory B-cells detected in the peripheral blood between 5 months and 12 months for each study group.**

|  | | **MenC** | **Diphtheria** | **Tetanus** |
| --- | --- | --- | --- | --- |
| **1-dose CRM group** | Mean difference | 0.63 | 0.65 | 0.70 |
|  | 95% CI | 0.45 to 0.80 | 0.44 to 0.89 | 0.51 to 0.89 |
|  | P-value | **<0.0001** | **<0.0001** | **<0.0001** |
| **2-dose CRM group** | Mean difference | 0.21 | 0.94 | 0.76 |
|  | 95% CI | 0.01 to 0.41 | 0.71 to 1.17 | 0.54 to 0.98 |
|  | P-value | **0.04** | **<0.0001** | **<0.0001** |
| **Control** | Mean difference | 0.26 | 0.71 | 0.62 |
|  | 95% CI | -0.01 to 0.54 | 0.39 to 1.03 | 0.32 to 0.92 |
|  | P-value | 0.06 | **<0.0001** | **<0.0001** |
| **1-dose TT group** | Mean difference | 0.35 | 0.89 | 0.63 |
|  | 95% CI | 0.12 to 0.57 | 0.62 to 1.15 | 0.38 to 0.88 |
|  | P-value | **0.003** | **<0.0001** | **<0.0001** |

95% CI: 95% confidence interval around the difference in means

P-values are reported for the comparison of the mean difference with 0

1-dose CRM: 1 dose MenC-CRM_197_ at 3 months of age; 2-dose CRM: 2 doses of MenC-CRM_197_ at 3 and 4 months of age; Control: No MenC primary vaccine doses; 1-dose TT: 1 dose MenC-TT at 3 months of age
